# Understanding the Role of Rutile TiO_2_ Surface Orientation on Molecular Hydrogen Activation

**DOI:** 10.3390/nano9091199

**Published:** 2019-08-26

**Authors:** Baohuan Wei, Frederik Tielens, Monica Calatayud

**Affiliations:** 1 Sorbonne Université, CNRS, Laboratoire de Chimie Théorique, LCT, F. 75005 Paris, France; 2 General Chemistry (ALGC), Materials Modelling Group, Vrije Universiteit Brussel (Free University Brussels-VUB), Pleinlaan 2, 1050 Brussel, Belgium

**Keywords:** hydrogen activation, rutile TiO_2_, hydrogen transfer

## Abstract

Titanium oxide (TiO_2_) has been widely used in many fields, such as photocatalysis, photovoltaics, catalysis, and sensors, where its interaction with molecular H_2_ with TiO_2_ surface plays an important role. However, the activation of hydrogen over rutile TiO_2_ surfaces has not been systematically studied regarding the surface termination dependence. In this work, we use density functional theory (PBE+U) to identify the pathways for two processes: the heterolytic dissociation of H_2_ as a hydride–proton pair, and the subsequent H transfer from Ti to near O accompanied by reduction of the Ti sites. Four stoichiometric surface orientations were considered: (001), (100), (110), and (101). The lowest activation barriers are found for hydrogen dissociation on (001) and (110), with energies of 0.56 eV and 0.50 eV, respectively. The highest activation barriers are found on (100) and (101), with energies of 1.08 eV and 0.79 eV, respectively. For hydrogen transfer from Ti to near O, the activation barriers are higher (from 1.40 to 1.86 eV). Our results indicate that the dissociation step is kinetically more favorable than the H transfer process, although the latter is thermodynamically more favorable. We discuss the implications in the stability of the hydride–proton pair, and provide structures, electronic structure, vibrational analysis, and temperature effects to characterize the reactivity of the four TiO_2_ orientations.

## 1. Introduction

Titanium oxide (TiO_2_) has been widely used in numerous fields, from everyday applications (paint, inks, toothpaste, makeup) to technological devices, such as dye-sensitized solar cells (DSSCs) [1,2], photoelectrochemical cells [3], photocatalysts [4], catalysis [5,6], sensors [7,8], biomedical treatments [9], lithium ion batteries [10], or photovoltaics [11,12]. The interaction of hydrogen with TiO_2_ surfaces plays an important role in many reaction processes [13,14,15,16,17,18,19,20] and has been widely studied [21,22,23,24,25,26,27]. Despite the high interest generated by hydrogen-titania interfaces, the nature of the species involved is still poorly understood—protons are generally reported as being stable in hydrogenated rutile (110) [28], atomic surface hydrogen has been found to prevent electron-hole recombination on an Au-TiO_2_ photocatalyst [14], and very recently hydride species have been characterized as being stable on its surface [29,30]. In this work, we investigate the role of the surface termination in the H_2_ dissociation and migration on rutile surfaces. We focus on the characterization of the stability of surface Ti-H species formed by interaction with H_2_ and their subsequent transfer to neighboring oxygen sites in order to provide a comprehensive picture of the adsorption, desorption, and diffusion mechanisms occurring at H-TiO_2_ interfaces.

In recent years, H_2_ dissociation over metal oxides has attracted great interest [31,32,33,34,35,36,37,38]. Two main mechanisms are proposed: homolytic and heterolytic dissociation [39]. It is widely thought that non-reducible metal oxides follow the heterolytic pathway forming MH/OH pairs, while reducible metal oxides proceed homolytically forming OH/OH pairs together with the metal site reduction. However, in recent years deviations from this rule have been proposed to explain experimental observations. Thus, García-Melchor et al. and Fernandez-Torre et al. reported that H_2_ dissociation on CeO_2_ (111) follows a heterolytic path, with H being transferred from Ce to a neighboring O, generating the homolytic product [34,40]. Chen and Pacchioni reported that on nanostructured MgO (001), the dissociation pathway depends on the choice of the support—on MgO/Ag (001), the heterolytic pathway is preferred, while with Au support, it follows the homolytic dissociation [31]. Very recently, Liu et al. reported the surface characteristics of anatase TiO_2_ after reduction with H_2_. In their study, they proposed that H_2_ can dissociate on oxygen vacancies—one H atom binds with a Ti to form the Ti-H bond, whereas the other one bonds with O to form Ti–OH [41]. Moreover, Hu et al. reported H_2_ dissociation on three TiO_2_ polymorphs [35], which showed that homolytic activation barriers are all high (1.48–1.68 eV), with rutile showing the highest activity.

It is well known that the surface properties strongly vary with different crystallographic orientations, which can greatly affect their reactivity [42,43,44,45,46]. For rutile TiO_2_, the main exposed low energy surface is the (110) surface, which is also the most studied [23,28,47,48,49,50,51]. There are also other terminations of rutile TiO_2_ that are experimentally accessible, such as (100) facet [52,53,54,55], (001) facet [56,57,58,59,60,61,62], (101) facet [8,60,63,64], and (011) facet [65,66,67]. Herein, we systematically study the hydrogen dissociation over four rutile TiO_2_ facets (001), (100), (110), and (101) by using density functional theorywith PBE+U(Perdew–Burke–Erzenhof functional with the Hubbard U correction). We consider a two-step mechanism for H_2_ dissociation: first, heterolytic dissociation to form TiH/OH pairs, and second, H transfer from Ti to O to form OH, accompanied by a two-electron transfer of the hydride to the Ti sites. We provide the structures of the reaction intermediates, the energetic profile of the two steps, the electronic structure of the systems involved, and the temperature effects to evaluate the barriers at room temperature for stoichiometric slab models. Vibrational frequencies for TiH and OH are also reported as a guide to identify relevant species on the different terminations.

## 2. Materials and Methods

**Figure 1:**
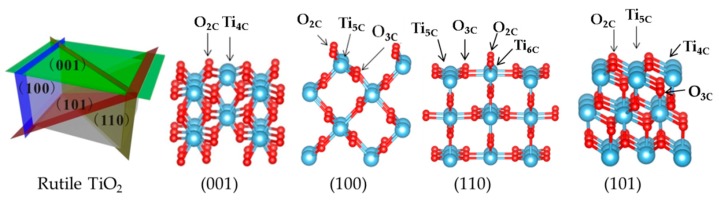
Side views of rutile TiO_2_ (001), (100), (110), and (101) surfaces. Note: Ti, blue; O, red.

Density functional theory (DFT) calculations were performed using the Vienna ab initio simulation package (VASP) version 5.4.4 [68]. Projector-augmented wave (PAW) pseudopotential was used to describe the core electron representation with 1, 4, and 6 valence electrons for H, Ti, and O, respectively [69,70]. The generalized gradient approximation (GGA) approach was used for the exchange and correlation potential with the Perdew–Burke–Erzenhof (PBE) functional [71,72]. The GGA+U approach of Dudarev et al. was used to treat the 3d orbital electrons of Ti with the effective Hubbard on-site Coulomb interaction parameter (U’ = U − J) [73]. We chose U’ = 4 according to the proposed value from previous works [24,28,74], referred herein as *U*. A 400 eV cutoff energy for the plane-wave basis set was found to correctly treat the rutile surface [28]. The dissociation of hydrogen on rutile TiO_2_ surfaces was investigated in the 1 × 1 unit cell for (001), (100), and (101) and in the 2 × 2 unit cell for the (110) surface. The open shell systems were treated with spin polarized calculations. The energy convergence was set to 3.0 × 10^−2^ eV for the ionic loop and 1.0 × 10^−4^ eV for the electronic loop. The slab models were cut from the optimized structure of bulk rutile (Figure 1). A vacuum layer of 20 Å was employed. The slab thickness used is given in Table 1. The lower-half layers of the slab were kept frozen and the upper-half layers were allowed to relax. We used the Monkhorst−Pack scheme to sample the Brillouin zone, and the distance between each k-point was 0.033 Å^−1^ [18,35]. The constrained minimization and climbing-image nudged elastic band (CI-NEB) methods were used to locate transition states (TS) [75,76]. In this work, the minimum energy pathway for each elementary reaction was discretized by a total of four images between the initial and final states. The imaginary frequency of every transition state was checked to connect initial and final states. The zero point energy (ZPE) vibration energy was calculated from vibrational frequencies as one-half of the sum of real-valued harmonic vibrational frequencies [77].

We also consider the effect of temperature by calculating the Gibbs free energy at room temperature (298 K); in the solid system, the pressure volume term *pV* can be ignored, thus:

*G*(*T*) = *H* − *TS* = *U* + *pV* − *TS* ≈ *U*(*T*) − *TS*(*T*) (1)

It is reasonable to only consider the vibrational contributions, therefore:

*U*(*T*) = *E_DFT_* + *E_ZPE_* + *U_vib_*(*T*) (2)

*S*(*T*) = *S_vib_*(*T*) (3)

For vibrational spectra, the density-functional perturbation theory (DFPT) linear response approach was used [78,79]. The matrix of Born effective charges (BEC) is obtained and indicates the change of involved atom’s polarizabilities. The infrared intensity can be described as in the following formula containing Born effective charges and the eigenvectors $e_{\beta }(s|\upsilon )$:

(4) $$f(\upsilon )={\sum_{\alpha }|\sum_{s\beta }Z_{\alpha \beta }^{*}(s)e_{\beta }(s|\upsilon )|}^{2}$$

where *α* and *β* are Cartesian polarization, $e_{\beta }(s|\upsilon )$ indicates the normalized vibrational eigenvector, and $Z_{\alpha \beta }^{*}$ indicates the effective charge tensor. To assess how the frequencies obtained depend on the computational setting, the performance of four different density functionals (PBE, Local Density Approximation (LDA), Perdew-Wang (PW91), Perdew-Burke-Ernzerhof revised for solids (PBESOL)), cut-off (300, 400, 500, 600, and 700 eV), choice of U (3, 4, 5, 6, and 7 eV), and the inclusion of dipole corrections were tested (see Supplementary Tables S1–S4 and Figures S7–S10). Although the numerical values are affected by computational settings, the trends between the different orientations are maintained.

Dispersion effects were evaluated for the heterolytic path for the intermediates and TS1 (the latter as a single-point calculation) by means of dispersion corrections (Grimme D3) (zero) [80] and the results are displayed in Supplementary Table S7 and Figure S12. Dispersion corrections were found to slightly stabilize adsorbates with respect to the non-corrected calculation and significantly decrease the barriers. However, they did not significantly alter the trends of the terminations, nor the vibrational frequencies (Supplementary Table S7 and Figure S12).

No dipole correction was used to account for the asymmetry of the slabs in the perpendicular direction. As our work is mainly based on the comparison of terminations, and as all of them should be affected in a similar manner by the spurious dipole, we do not expect it to have a significant impact on the conclusions. As can be seen in Supplementary Table S6 and Figure S11, the inclusion of dipole corrections did not have a significant effect in the vibrational frequencies.

### Slab Model

We optimized the bulk TiO_2_ rutile unit cell obtaining values of a = b = 4.661 Å and c = 2.961 Å, in agreement with experimental parameters of a = 4.593 Å and c = 2.958 Å [23]. The calculated lattice parameters for bulk rutile TiO_2_ were overestimated by 1.46% for a and only 0.10% for c with respect to the experimental value, and the optimized values were used to build the slab models.

The four rutile TiO_2_ surface (001), (100), (110), and (101) stoichiometric terminations are represented in Figure 1 and the main structural parameters are reported in Table 1. As we can see, the facets (001) and (110) are roughly flat, while (100) and (101) facets are uneven. On the surfaces, the coordination number of titanium sites vary from 4 to 6(001) has only Ti_4C_; (101) possesses Ti_4C_ and Ti_5C_; (100) has only Ti_5C_; and (110) has Ti_5C_ and Ti_6C_. Regarding oxygen, the surface coordination varies from two- to three-fold—(001) has only O_2C_, while the other three exhibit O_2C_ and O_3C_. The surface energy E_surf_, calculated as the difference in energy between the slab and the bulk divided by twice the area, follows the trend of coordination—the lower the surface atomic coordination, the higher the surface energy. Thus, (001), where Ti and O are poorly coordinated, shows E_surf_ 1.30 J nm^−2^, whereas (110), where the atoms are more coordinated, shows E_surf_ 0.55 J nm^−2^.

## 3. Results and Discussion

### 3.1. H_2_ Dissociation

**Figure 2:**
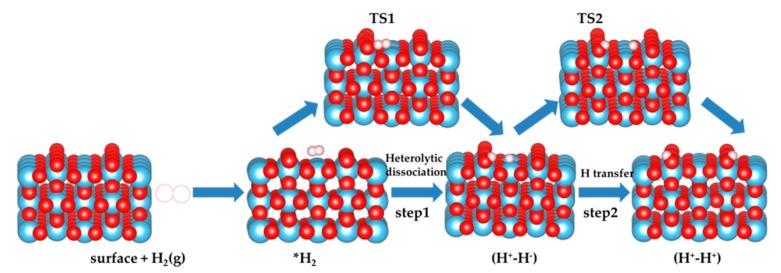
Schematic two-step mechanism considered in the present work. The heterolytic dissociation pathway of H_2_ over TiO_2_ surface (step 1) and sequential H transfer from Ti to near O (step 2). Ti, O, and H atoms are depicted by blue, red, and white spheres, respectively.

**Figure 3:**
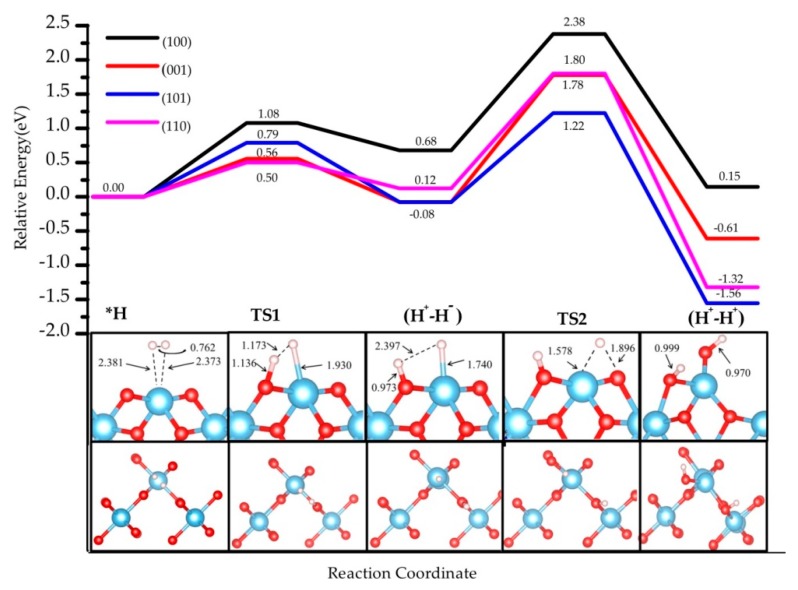
The energy profile of hydrogen dissociation and H transfer from Ti to near O on four rutile TiO_2_ surfaces, namely (001), (100), (101), and (110). Inset images show the pathway (side view and top view) on TiO_2_ (001); the three other pathways are depicted in Supplementary Figures S1–S3. The bond distance is in Å.

Firstly we investigated the heterolytic pathway for H_2_ dissociation on the four selected rutile TiO_2_ selected. In the first step, the H_2_ molecule physisorbs on the surface forming the adduct H_2_*. Then, the heterolytic H_2_ dissociation takes place between the Ti site and a neighboring O atom through a transition structure (TS1), generating a pair of O−H and Ti−H bonds (H^+^-H^−^ species). The second step involves the transfer of the hydride (H^−^) on the Ti site to a nearby O, leading to 2 O-H hydroxyl groups (H^+^-H^+^) and a two-electron transfer to surface titanium sites that become reduced. The transition state associated with this step is labeled as TS2. The reaction pathway involving these two steps is schematized in Figure 2, and the calculated energies are reported in Table 2. The energy profile of H_2_ dissociation over the four surfaces is shown in Figure 3.

Here, all adsorption energies are referred to the energy of physisorbed TiO_2_-H_2_. The path for the TiO_2_ (001) surface is illustrated, and those corresponding to the other three terminations are provided in Supplementary Figures S1–S3. Several pathways were considered involving different surface sites. For the heterolytic step, on (001) there is a unique possible pathway with only one kind of O_2c_ site and one Ti_4c_ site on the surface. On (100), besides the pathway reported in Supplementary Figure S1, there is also one additional combination of Ti_5C_ and O_2C_ sites (Supplementary Figure S6a), in which the direction of the OH bond is almost perpendicular to the direction of the TiH bond, which makes this combination less stable than the one selected. For (101), two other possible structures involving Ti_5C_ and O_2C_ (Supplementary Figure S6b) and Ti_4C_ and O_3C_ (Supplementary Figure S6c) resulted in less stable systems than the one retained. The model structures retained are stabilized as a consequence of the saturation of poorly coordinated sites of Ti_4C,_ Ti_5C_, and O_2C_ upon hydrogenation, and in some cases the formation of hydrogen bonds.

The stability of the (H^+^, H^−^) intermediate is slightly exothermic for the (001) and (101) terminations (−0.08 eV), whereas it is slightly endothermic for the (110) by 0.12 eV, and for the (100) by 0.68 eV. Hydrogen bonds between TiH and OH species form in all the terminations except (101). Whereas the terminations showing the poorest coordination exhibit the most exothermic adsorption energy for the (H^+^, H^−^) intermediate, the most highly coordinated slabs show less exothermic values. However, the most highly coordinated (110) slab exhibits a significantly lower adsorption energy than (100). The activation barriers of heterolytic H_2_ dissociation on the four TiO_2_ surfaces follow the trend (110) 0.50 eV < (001) 0.56 eV < (101) 0.79 eV < (100) 1.08 eV. As for the adsorption energy, a trend appears between coordination and kinetic barriers for (001), (101), and (100), whereas (110) presents lower values than expected (its higher coordination should lead to the most endothermic values). Our results are consistent with previous studies. For the (001) surface, our activation energy (0.56 eV) is consistent with the one reported previously (0.68 eV) [14]. The difference comes from the use of a different Hubbard parameter (U = 7 eV) and unit cell (2 × 1). Our activation energy for the (110) surface, 0.50 eV, is larger than the 0.37 eV reported for a much narrower slab (3-TiO_2_-layer thick slab model [34]), highlighting the important role of slab thickness in the construction of a model.

**Figure 4:**
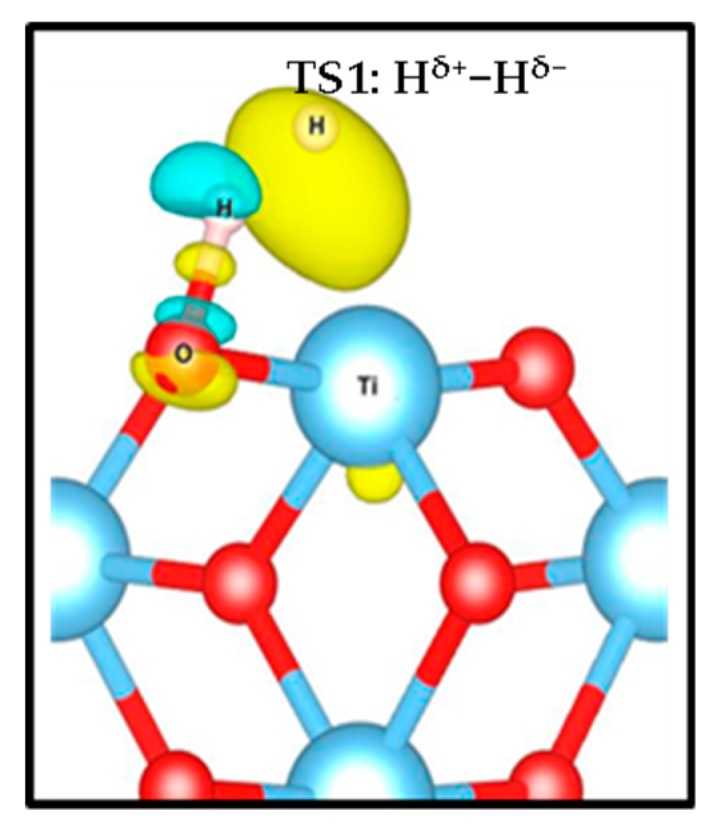
Charge density difference of transition state 1(TS1)illustrating the formation of the H^δ+^-H^δ−^ tight ion pair on the (001) surface. Yellow and green iso-surfaces show an electronic density gain and depletion, respectively.

According to our results, the (H^+^, H^−^) intermediate is more likely to be formed on (001) and (101) terminations, however the poor stability and the low barriers could induce the inverse reaction, i.e., the recombination and desorption as H_2_ (see below). These results suggest that the rutile TiO_2_ (001) exhibits the most likely H_2_ heterolytic dissociation path in the series, with the lowest activation energy and a slight stabilization of the product. This specific reactivity could be associated with the low coordination of the surface titanium site—the four-fold coordinated Ti site in the (001) termination stabilizes the hydride Ti-H species to increase the number of neighbors. In the transition structure 1 (TS1) displayed in Figure 3, the Ti-H distance is 1.93 Å (1.74 Å in the intermediate), and the species appears in interaction with the OH group (H-H distance of 1.17 Å) with an imaginary frequency of 992.39 cm^−1^. The charge density difference analysis shows that there exists a tight ion pair in TS1 where the H on Ti gains electronic density and the H on O is deprived, forming a H^+^-H^−^ pair (Figure 4). This is consistent with a moderate polarization of the H_2_ moiety, as shown in the Bader analysis discussed below. In this TS1 structure, the four atoms involved Ti-H…H-O are coplanar.

For the other three facets, similar structures are found for TS1 involving coplanar Ti-H…O-H geometries. For the (100) termination, the transition state of this dissociation process shows −1106.91 cm^−1^ Ti-H vibration mode, with 1.83 Å for Ti−H and 1.10 Å for the H-H distance. For the (110) and (101) facets, the Ti-H vibration modes are −704.20 cm^−1^ and −1289.80 cm^−1^, respectively. The Ti-H bond distances are both 1.95 Å, and the O-H distances are 1.22 Å and 1.33 Å, respectively.

The second step in the mechanism is the transfer of the H^−^ on the Ti site to the nearest two-fold-coordinated O site, finally yielding two hydroxyls on the surface and a reduction of the Ti sites. The final products (H^+^-H^+^) are thermodynamically the most stable ones in the path: (001) −0.61 eV, (110) −1.56 eV, (101) −1.09 eV, and (100) 0.15 eV. H-bonds are formed in some of the structures, which results in larger stabilization. In the cases of (001) and (100), the final products involve a rearrangement of the surface bonds—a Ti-O-Ti breaks to form a Ti-OH moiety. The activation barriers are significantly higher than for the first step, ranging from 1.30 to 1.80 eV. These results indicate an unfavorable evolution to the homolytic product from the heterolytic intermediate. Thus, the hydride TiH species could be kinetically stabilized on TiO_2_ surfaces with a possible recombination to regenerate and desorb H_2_ at low temperatures, whereas the reduction step would require much higher energies to occur. Nevertheless, the most thermodynamically stable product is found for step 2 and involves the presence of two hydroxyl groups and two Ti^3+^ sites; the latter originate from the electron transfer from the hydride to two titanium sites. This transfer results in open-shell systems that can be characterized by the presence of two unpaired electrons. 

We have looked for correlations between adsorption energy, barrier heights, and geometry (TiH, HH, and OH distances), as well as Bader charges in TSs, and our results indicate no clear relationship. This is very interesting, as for CeO_2_ those correlations do appear [81]. This might point to an ionocovalent character of Ti-O bond compared to the more ionic Ce-O bond, which would facilitate the formation of the H^+^-H^−^ ionic pair, or to the important role of the local topology in stabilizing intermediates and transition structures. As a general trend, the activation barriers seem related to the coordination numbers of Ti and O on the surfaces, with the (110) termination behaving in a different way than is expected from its highly coordinated surface sites.

### 3.2. Electronic Structure

**Figure 5:**
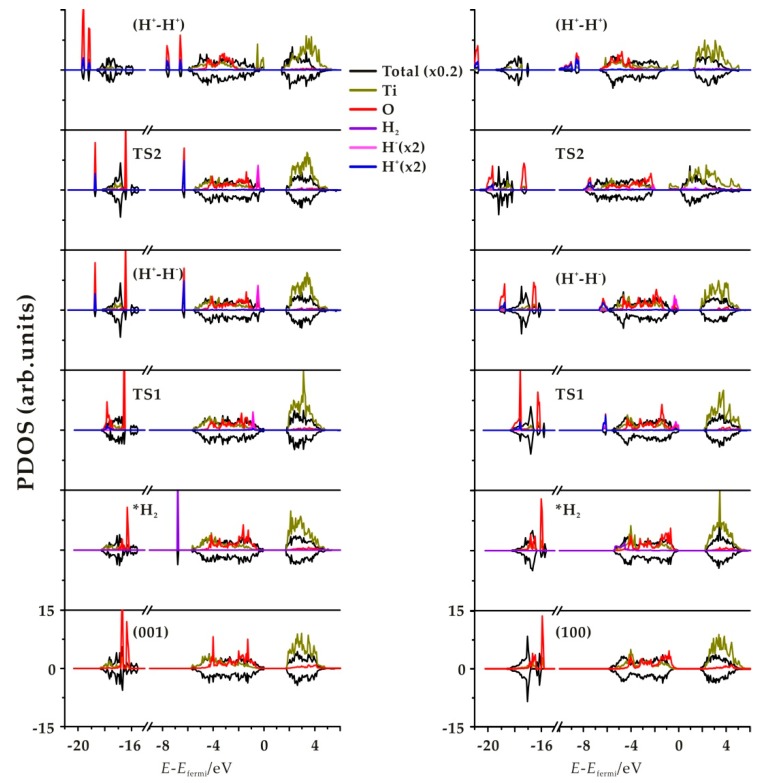
Total and projected densities of state (PDOS) of the TiO_2_ slab, *H_2_, TS, *(H^+^, H^−^), and *(H^+^-H^+^) for the (001) (**left**) and (100) (**right**) surfaces. For the PDOS, only the Ti and O involved in the two processes are projected. Positive *density of states* (DOS) correspond to spin up and negative to spin down.

**Figure 6:**
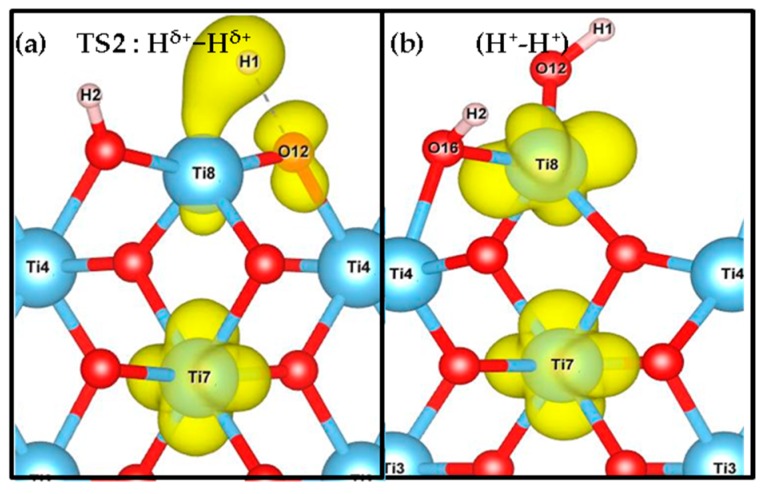
Spin density of TS2 (**a**) and (H^+^-H^+^) species (**b**) indicating the distribution of unpaired electrons on the (001) facet. The spin densities for TS2 and (H^+^-H^+^) species on the other three facets are shown in Supplementary Figure S5.

In order to characterize in more detail the electronic structure of the structures involved in the hydrogenation mechanisms, we have computed the *density of states* (DOS); Figure 5 and Supplementary Figure S4) for step 1 and step 2 of the four terminations considered. As unpaired electrons are involved, especially in H transfer process step 2, spin up and spin down are represented. The features of these four facets are similar and only the (001) and (100) facets are displayed in Figure 5. The other two facets are shown in Supplementary Figure S4. At the bottom of the plot a hydrogen molecular band appears as a sharp narrow peak in the valence region due to H_2_ physical adsorption. In TS1, we observe a splitting in two bands associated with H^+^ and H^−^ species that overlap with the slab levels. For the product of heterolytic dissociation (H^+^-H^−^), the H^−^ band is the highest occupied energy level, with a sharp peak at the Fermi level. For the TS2 of subsequent H transfer from Ti to nearby O, there still exists one H^+^ band and one H^−^ band, but the intensity decreases. The existence of wide, weak peaks in the gap indicates an early reduction of the Ti site in TS2 on (100) and (110) facets, while no corresponding peak appears on (001) and (101). For the H transfer process product (H^+^-H^+^) species, we observe the H^+^ levels corresponding to OH groups in O-H bonds in the valence band, which appear as two distinct peaks if they correspond to inequivalent hydroxyl groups. Also, Ti states appear in the gap below the Fermi level, indicating the reduction of the Ti sites. This is consistent with the picture of the spin density plots (Figure 6 and Supplementary Figure S5), indicating that the unpaired electrons from the hydride transfer are trapped by two Ti ions that get reduced, confirming the nature of Ti^3+^ sites. Note that the approach used in the present work does not allow one to state unambiguously which Ti sites are reduced—it only confirms qualitatively that two distinct Ti sites are involved.

Bader charge analysis [82] was carried out to complement the characterization of the electronic structure of the systems studied. In Table 3, we can follow the electronic charges during the two processes, whereas Table 4 shows the Bader analysis for the spin density. In step 1, the adsorbed hydrogen species shows a slightly polarized H-H bond. In TS1, the H-H bond is more polarized, generating a tight ion pair with charges in the range 0.35-0.48 |e| for the H^+^ and −0.31 to −0.41 |e| for the H^−^ species. The intermediate (H^+^, H^−^) species is characterized by charges in the range 0.65–0.70 |e| and −0.30 to −0.41 |e| for H^+^ and H^−^, respectively. Moreover, the oxygen involved in this hydrogen transfer process shows electron gain of about +0.15–0.30|e| compared to the same O in the slab. The Bader charge of the products (H^+^-H^+^) show values from 1.78 to 1.87 |e| for the surface Ti sites carrying the electrons. Actually, based on our spin density results (see Figure 6, Supplementary Figure S5 and Table 4) two Ti are reduced for every facet. For the TS2, one of the Ti on the surface partially decreases its positive charge, indicating partial reduction. Finally, in the H^+^-H^+^ species the two H are characterized as protons, whereas two Ti sites decrease their positive charge, indicating that they host the reduction electrons, and the integrated spin density varies from 0.90 to 1.05 |e| (See Table 4). It is worth stating that the O site involved in the H transfer process also contains a small amount of unpaired electrons of 0.24 |e| for TS2 of both (001) and (101) facets, as can be seen in Figure 6 for the (001) case.

### 3.3. Effect of the Hubbard Correction U

**Figure 7:**
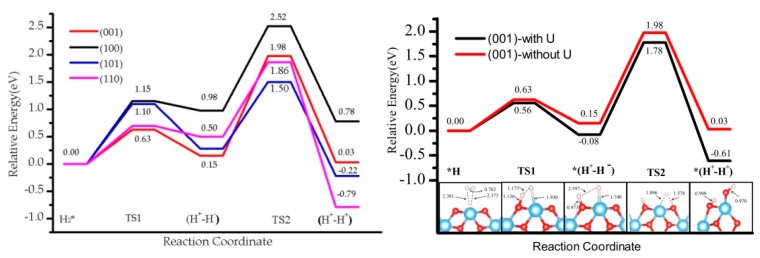
Comparison of energy profile on four facets without U correction (**left**). Comparison of energy profile on (001) when U is 4.0 eV and without U (**right**).

The values without U correction were considered to analyze the effect of U in the energetic profile, which is reported in Table 2. The profile is similar to the one obtained for U = 4 eV (see Figure 7 for the (001) case and Supplementary Materials for the others). In step 1, the heterolytic dissociation leads to (H^+^-H^−^) products stable at 0.15 eV (001), 0.28 eV (101), 0.98 eV (101), and 0.50 eV (110), and barriers of 0.63 eV, 1.10 eV, and 1.15 eV, 0.70 eV, respectively, which is ~0.20 eV higher in energy than for the U = 4 eV case (Table 2, Figure 3). The increase in the values is significantly higher in step 2, where the (H^+^-H^+^) product is higher in energy by ~0.60 eV in the absence of U correction, and is associated with the stabilization of the localized solution favored by the U = 4 eV term with respect to the U = 0 eV case. In general, the activation barriers are not significantly affected by the U value, with the exception of (110) and (100) in step 1 (formation of H^+^-H^−^), where the U = 0 eV leads to a TS1 very close in energy to the H^+^-H^−^ intermediate. The backward reaction i.e., recombination desorption of H_2_, would thus be barrierless and the intermediate would not be stable at all. The overall profile and the trend of the activity for H_2_ dissociation and subsequent H transfer for the four TiO_2_ surfaces is maintained.

### 3.4. Vibrational Spectrum

**Figure 8:**
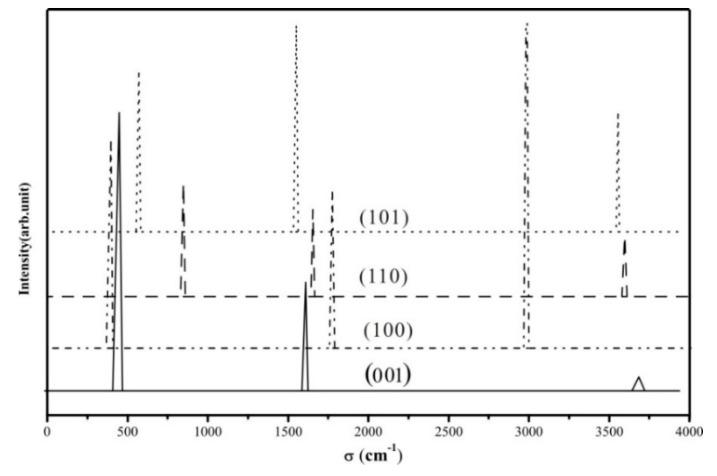
Computed IR spectra of the TiO_2_-(H^+^, H^−^) species for the four selected terminations. Intensities are given in arbitrary units.

**Figure 9:**
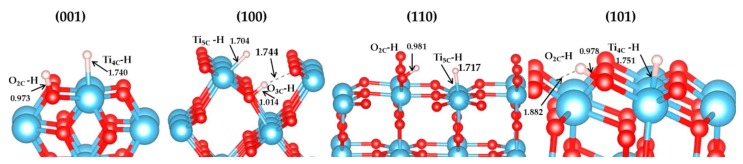
Structures of (H^+^-H^−^) species on (001), (100), (110), and (101) surfaces (bond distance in Å).

We computed the vibration frequency and IR spectra of H_2_ heterolytic dissociation products (H^+^-H^−^) for the four terminations. No scaling factor was applied. The vibration modes are shown in Table 5 and Figure 8, and present three main regions: Ti–H and O–H bending modes at low frequencies (below 1000 cm^−1^); the vibration frequencies of Ti–H lie in the range of 1500–1800 cm^−1^; the stretching OH modes are characterized by higher frequencies (between 2900 cm^−1^ and 3800 cm^−1^). Ti–H stretching modes of the four species are seen in the calculated spectrum at 1644 cm ^−1^ (001), 1768 cm^−1^ (100), 1653 cm^−1^ (110), and 1577 cm^−1^ (101), corresponding to the expected Ti–H IR spectral region (around 1600 cm^−1^) [83].The hydrides of (001) and (101) facets are Ti_4C_-H, while they are Ti_5c_-H on (100) and (110) surfaces, as displayed in Figure 9. Previous studies using electron-stimulated desorption (ESD) [84] and low-energy ion scattering (LEIS) [85] reported that the annealed TiO_2_ surface is compensated by H, which is bonded in the Ti–H as well as O–H with bridging O or a subsurface, but no specific frequencies were provided. Recently, Yan et.al indicated the formation of Ti-H species on the P25 TiO_2_ surface [86].

For the (O-H) vibrations, there are many experimental reports by various authors (see selected ones in Supplementary Table S5), showing that the vibrations can be greatly influenced by the nature of the site, the surface topology, the presence of defects and coverage [87], as well as the polymorph [88]. In the present work, we perform an analysis of OH vibrations for the four terminations considered (see Table 5, Figure 9) as a guide for qualitative assignment. It is found that the OH stretching vibrations are different between these four facets. The calculated IR results of TiO_2_ (100) surface (2976.54 cm^−1^) correspond to a Ti_5C_-O_3C_H exhibiting an H bond with one O site nearby. An experimental value of 3550 cm^−1^ for OH on (100) [89] was reported for the adsorption of water on the surface, most likely assigned to terminal hydroxyl groups. Our value is consistent with a higher coordination of the hydroxyl group (three-fold in our case), as well as with the presence of a hydrogen bond, both blue-shifting the vibration with respect to the experimental value. For the TiO_2_ (101) surface (3622.37 cm^−1^) it corresponds to a Ti_4C_-O_2C_H. Experimentally, the OH stretching vibrations from water adsorption are observed at 3680 and 3610 cm^−1^ [89]. For the TiO_2_ (110) surface (3606.59 cm^−1^) the vibration corresponds to Ti_6C_-O_2C_H, which is lower than in a previous theoretical study by Wöll (3700 cm^−1^) [90]. Note that the model used in the work of Wöll et al. involves a hydroxyl perpendicular to the slab, whereas in our work the hydroxyl is tilted. Other experimental works report 3665 and 3690 cm^−1^ measured by High-Resolution Electron Energy Loss Spectroscopy (HREELS) [91,92], and 3711 cm^−1^ by IR [93] on systems obtained by H_2_O adsorption on a clean single-crystal TiO_2_ surface.

As a general remark, the lack of experimental data in well-controlled structures and conditions make an assessment of the vibrational spectra of surface hydroxyl and hydride species difficult, although several trends can be observed. First, the vibrations are dependent on the surface topology due to specific local chemical environments. Second, the coordination of oxygen and titanium sites seems to play a role, as well as hydrogen bonds formed between TiH/OH pairs and neighboring O sites. Overall, our results are consistent with previous experimental and theoretical data published in the literature and provide a set of spectra to stimulate the search of TiH/OH species on different rutile terminations.

### 3.5. The H_2_ Recombination-Desorption Reaction

**Figure 10:**
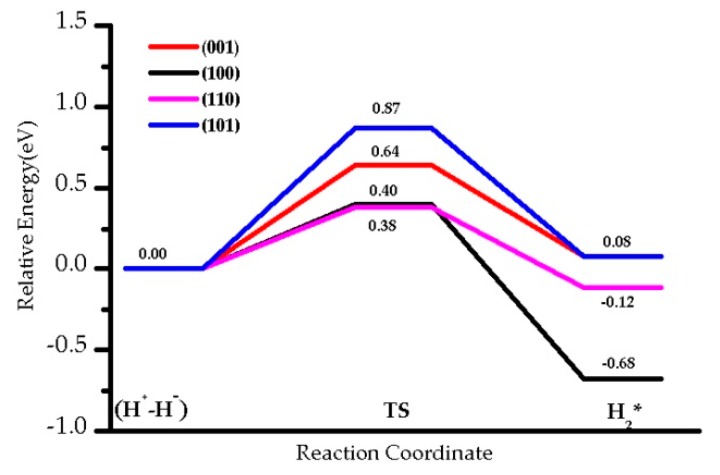
The energy profile of hydrides TiH (H^+^-H^−^) species recombination with OH to desorb H_2_ on four facets at 0 K, U = 4 eV.

We studied the energy barriers for hydride TiH/OH species recombination to regenerate and desorb H_2_ on four facets (Figure 10, Table 2). The corresponding barrier for that process, $E_{act}^{back}$, requires 0.64 eV for (001), 0.87 eV for (101), 0.38 eV for the (110), and 0.40 eV for the (100) slabs. The backward activation energies for the facets (001) and (101) are larger than those found for facets (110) and (100), probably due to the higher stability of the (H^+^-H^−^) species. Contrary to the dissociation process, the desorption of H_2_ is slightly endothermic for the (001) and (101) terminations (0.08 eV), whereas it is exothermic for the (110) by −0.12 eV, and for the (100) by −0.68 eV. On (101) and (001) facets, hydrogen dissociation, and therefore (H^+^-H^−^) formation, is slightly more favorable than H_2_ desorption: 0.79 eV vs. 0.56 eV for (101), 0.87 eV vs. 0.64 eV for (001). H_2_ dissociation and desorption occur with similar barriers on (110), with 0.50 eV and 0.40 eV, respectively. Thus, it is expected that the (H^+^-H^−^) intermediate involving Ti-H species is more likely to be observed in (001), and to a lesser extent (101), where the (100) and (110) would lead to recombination and desorption.

### 3.6. Zero-Point Energy Correction and Effect of Temperature

**Figure 11:**
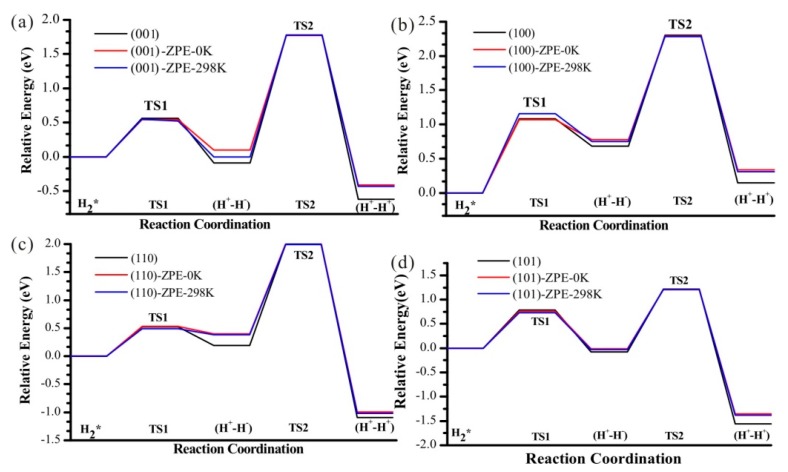
The heterolytic pathway of H_2_ dissociation and subsequent H transfer from Ti to O nearby on four (**a**–**d**) TiO_2_ facets, considering zero point energy (red lines). Blue lines indicate the profiles for T = 298 K.

The energy profiles with Zero Point Energy (ZPE) correction are also studied together with the Gibbs free energies for T = 298 K (Figure 11). With ZPE correction, the energy for these two steps increases, while it does not affect the kinetic barriers. Temperature has almost no effect on this reaction profile.

As a final remark, many other factors may have a deep influence on the behavior of TiO_2_ regarding hydrogenation—the presence of surface and subsurface defects [94], the nature of the bulk phase [95], nanostructuring [96,97], interfacial water [98], or reduction [99]. More fundamental works to elucidate the structure of hydrogenated surfaces are needed to build a robust scenario for the complex behavior observed [100].

## 4. Conclusions

The mechanisms of H_2_ dissociation on four different rutile TiO_2_ facets by means of density functional theory (PBE+U) calculations have been investigated. The results showed that the topology of the surface has a moderate effect on H_2_ dissociation on TiO_2_ kinetically and also thermodynamically. We found that for all four surfaces, the heterolytic dissociation pathway towards hydride–hydroxyl surface pairs is kinetically more favorable than the H transfer process towards substrate reduction, although the reduction product, with only surface hydroxyl groups, is thermodynamically more favorable. On (110) and (100), the hydride–hydroxyl pair formed can recombine and desorb as molecular dihydrogen, whereas the (001), and to a lesser extent (101), stabilize the hydride–hydroxyl pair. The energetics of the reaction seems related to the coordination numbers of Ti and O on the surfaces, although (110) shows a specific behavior. No clear trend relating adsorption energies and barriers with local geometry or charges was found. The electronic structure analysis allows characterization of charge and electron transfers. The IR spectra of the (H^+^-H^−^) pair species were also computed indicating the vibrational region of Ti-H species on TiO_2_ facets in the range of 1550–1750 cm^−1^. The frequencies are found to depend on the facet exposed and could be used as a qualitative guideline to identify them experimentally.

## Figures and Tables

**Table 1:** Size, composition, layers, and coordination numbers of atomic surface.

Surface	(001)	(100)	(110)	(101)
Supercell	1 × 1	1 × 1	2 × 2	1 × 1
Composition (TiO_2_ units)	8.00	8.00	32.00	8.00
TiO_2_ layers (frozen/relaxed)	8	8	4	8
	4/4	4/4	2/2	4/4
Coordination	O(2)	O(2,3)	O(2,3)	O(2,3)
	Ti(4)	Ti(5)	Ti(5,6)	Ti(4,5)
Parameter: *a*, *b* in Å	*a* = 4.661	*a* = 4.661	*a* = 6.018	*a* = 5.522
	*b* = 4.661	*b* = 2.962	*b* = 13.096	*b* = 4.661
Automatic k-point = 1/30 Å^−1^	5 × 5 × 1	5 × 8 × 1	4 × 2 × 1	5 × 5 × 1
E_surf_ (J nm^−2^)	1.30	0.73	0.55	1.07

**Table 2:** Reaction energy (∆E), dissociation activation energy ($E_{act}^{forw}$), and backward activation energies ($E_{act}^{back}$ eV) for step 1, and the H transfer barrier of step 2 (E*_act_*_2_). Values in brackets are energies without U correction (see below and Supplementary Information). All energies are referred to the physisorbed TiO_2_-H_2_ system. * indicates adsorption state.

	(001)	(100)	(110)	(101)
H_2_ *	0.00 (0.00)	0.00 (0.00)	0.00 (0.00)	0.00 (0.00)
TS1	0.56 (0.63)	1.08 (1.15)	0.50 (0.70)	0.79 (1.10)
(H^+^-H^−^)	−0.08 (0.15)	0.68 (0.98)	0.12 (0.50)	−0.08 (0.28)
TS2	1.78 (1.98)	2.38 (2.52)	1.80 (1.86)	1.22 (1.50)
(H^+^-H^+^)	−0.61 (0.03)	0.15 (0.78)	−1.32 (−0.79)	−1.56 (−0.22)
∆E_1_	−0.08 (0.15)	0.68 (0.98)	0.12 (0.68)	−0.08 (0.28)
$E_{act}^{forw}$	0.56 (0.63)	1.08 (1.15)	0.50 (0.70)	0.79 (1.10)
$E_{act}^{back}$	0.64 (0.48)	0.40 (0.17)	0.38 (0.20)	0.87 (0.82)
∆E_2_	−0.53 (−0.12)	−0.53 (−0.20)	−1.44 (−1.47)	−1.48 (−0.50)
E_*act*2_	1.86 (1.83)	1.70 (1.54)	1.68 (1.18)	1.40 (1.22)

**Table 3:** Bader charges (|e|) of H and involved Ti and O_a_ in the H_2_ dissociation process, and involved H, Ti, and O_b_ in subsequent H transfer from Ti to O process for H_2_ *, TS and (H^+^-H^−^), and (H^+^-H^+^). For step 1, the O involved was labeled O_a_, and O_b_ in step 2.

	qTi^+^ /qO^−^	qH^+^/qH^−^ qTi^+^/qO_a_^−^			qH^+^/qH^−^ qTi^+^/qO_b_^−^		qH^+^/qH^+^ /qTi^+^/qO_b_^−^
	Slab	H_2_ *	TS1	(H^+^-H^−^)-O_a_	(H^+^-H^−^)-O_b_	TS2	(H^+^-H^+^)
(001)	1.98/−1.00	0.02/−0.01 /1.98/−1.00	0.48/−0.41 /1.97/−1.02	0.67/−0.42 /1.95/−1.22	0.67/−0.42 /1.95/−0.98	0.63/−0.10 /1.90/−0.97	0.65/0.61 /1.79/−1.26
(100)	2.03/−1.07	0.04/−0.02 /2.01/−1.07	0.43/−0.32 /1.96/−1.12	0.65/−0.30 /1.90/−1.24	0.65/−0.30 /1.90/−0.98	0.64/−0.01 /1.77/−1.10	0.60/0.60 /1.78/−1.27
(110)	2.01/−0.90	0.04/−0.02 /2.04/−0.92	0.35/−0.31 /2.01/−0.97	0.70/−0.34 /1.93/−1.22	0.70/−0.34 /1.93/−0.91	0.67/0.00 /1.95/−0.92	0.64/0.62 /1.87/−1.15
(101)	1.99/−0.96	0.04/−0.03 /1.98/−0.96	0.38/−0.40 /1.97/−1.03	0.67/−0.40 /1.96/−1.20	0.67/−0.40 /1.96/−0.93	0.60/−0.10 /1.90/−1.25	0.60/0.66 /1.83/−1.30

**Table 4:** The number of unpaired electrons of TS2 and (H^+^-H^+^) species. Only involved atoms are shown.

Slab	(001)	(100)	(110)	(101)	Slab	(001)	(100)	(110)
Species	TS2	(H^+^-H^+^)	TS2	(H^+^-H^+^)	TS2	(H^+^-H^+^)	TS2	(H^+^-H^+^)
Total	1.70	2.00	1.90	2.00	1.92	2.00	1.70	2.00
Ti	0.80	0.97	0.99	0.91	0.24	0.90	0.80	0.99
Ti	0.16	1.00	0.11	1.05	0.24	0.90	0.16	0.99
O	0.24	0.00	0.02	0.00	0.00	0.00	0.24	0.00
H1	0.44	0.00	0.87	0.00	0.97	0.00	0.44	0.00
H_2_	0.00	0.00	0.00	0.00	0.10	0.00	0.00	0.00

**Table 5:** Computed IR wavenumbers (cm^−1^) and intensities (in brackets) of Ti−H and O−H stretching modes of (H^+^, H^−^) species for the four terminations studied.

Stretching Modes	(001)	(100)	(110)	(101)
(Ti-H)	1644.78 (0.39)	1768.74 (0.52)	1653.87 (0.32)	1577.45 (0.76)
(O-H)	3742.87 (0.05)	2976.54 (1.56)	3606.59 (0.22)	3622.37 (0.44)

## Supplementary Materials

The following are available online at https://www.mdpi.com/2079-4991/9/9/1199/s1. Figure S1: H_2_ dissociation on rutile TiO_2_ (100) facets with U = 4 eV and U = 0 eV. Figure S2: H_2_ dissociation on rutile TiO_2_ (110) facets with U = 4 eV and U = 0 eV. Figure S3: H_2_ dissociation on rutile TiO_2_ (101) facets with U = 4 eV and U = 0 eV. Figure S4: Projected Density of State (PDOS) of the TiO_2_ slab, H_2_*, TS, and (H^+^, H^−^), (H^+^-H^+^) for the (110) (left) and (101) (right) surfaces. Figure S5: Spin Density of TS2 and (H^+^-H^+^). Figure S6: Structures of (H^+^-H^−^) for the other adsorption modes considered. Tables S1–S6 and Figures S7–S11 IR frequencies. Tables S7 and S12: Grimme D3 corrections. Geometric structures for the intermediates and transition structures used in this work (POSCAR - VASP input files- format).
