# Supplementary material for: Understanding the Role of Rutile TiO2 Surface Orientation on Molecular Hydrogen Activation
Source: Nanomaterials (Basel). 2019 Aug 26;9(9):1199. doi: 10.3390/nano9091199 (PMC6780095; doi:10.3390/nano9091199)
Supplement: Supplementary file 1 [file nanomaterials-09-01199-s001.pdf]

Supporting Information

Understanding the role of Rutile TiO_2 Surface Orientation on Molecular Hydrogen Activation

Baohuan Wei [1], Frederik Tielens [2], Olivier Matz [2] and Monica Calatayud [2,*]

The effect of U

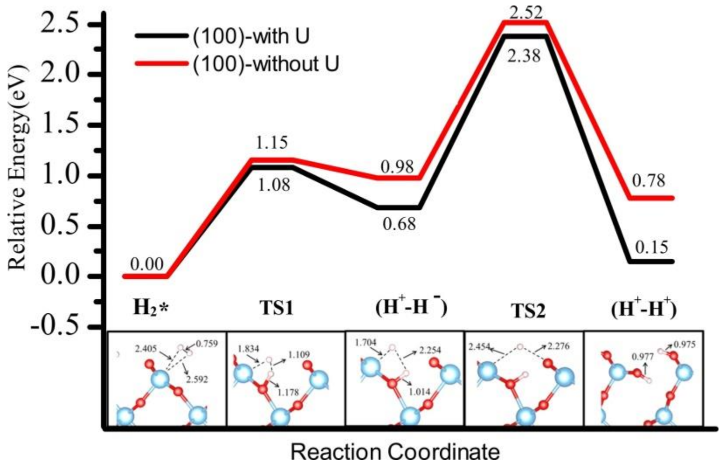

Figure S1. H_2 dissociation on rutile TiO_2 (100) facets with U= 4 eV and U = 0 eV.

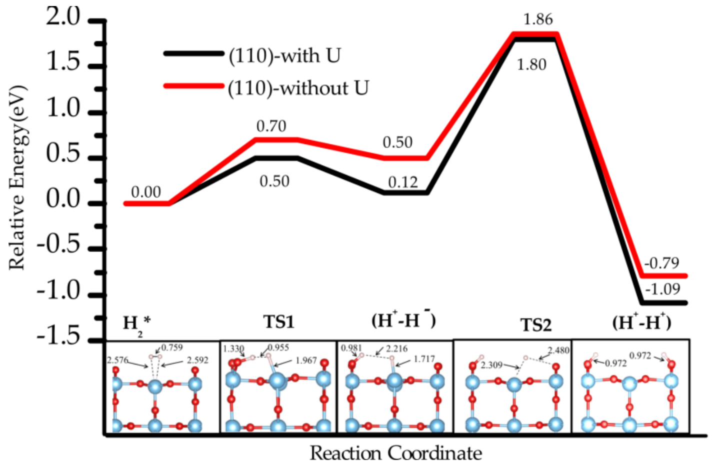

Figure S2. H_2 dissociation on Rutile TiO_2 (110) facets with U= 4 eV and U = 0 eV.

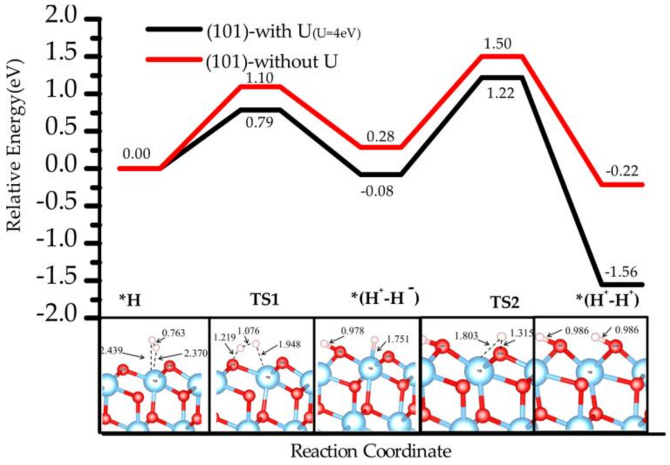

Figure S3. H_2 dissociation on Rutile TiO_2 (101) facets with U= 4 eV and U= 0 eV.

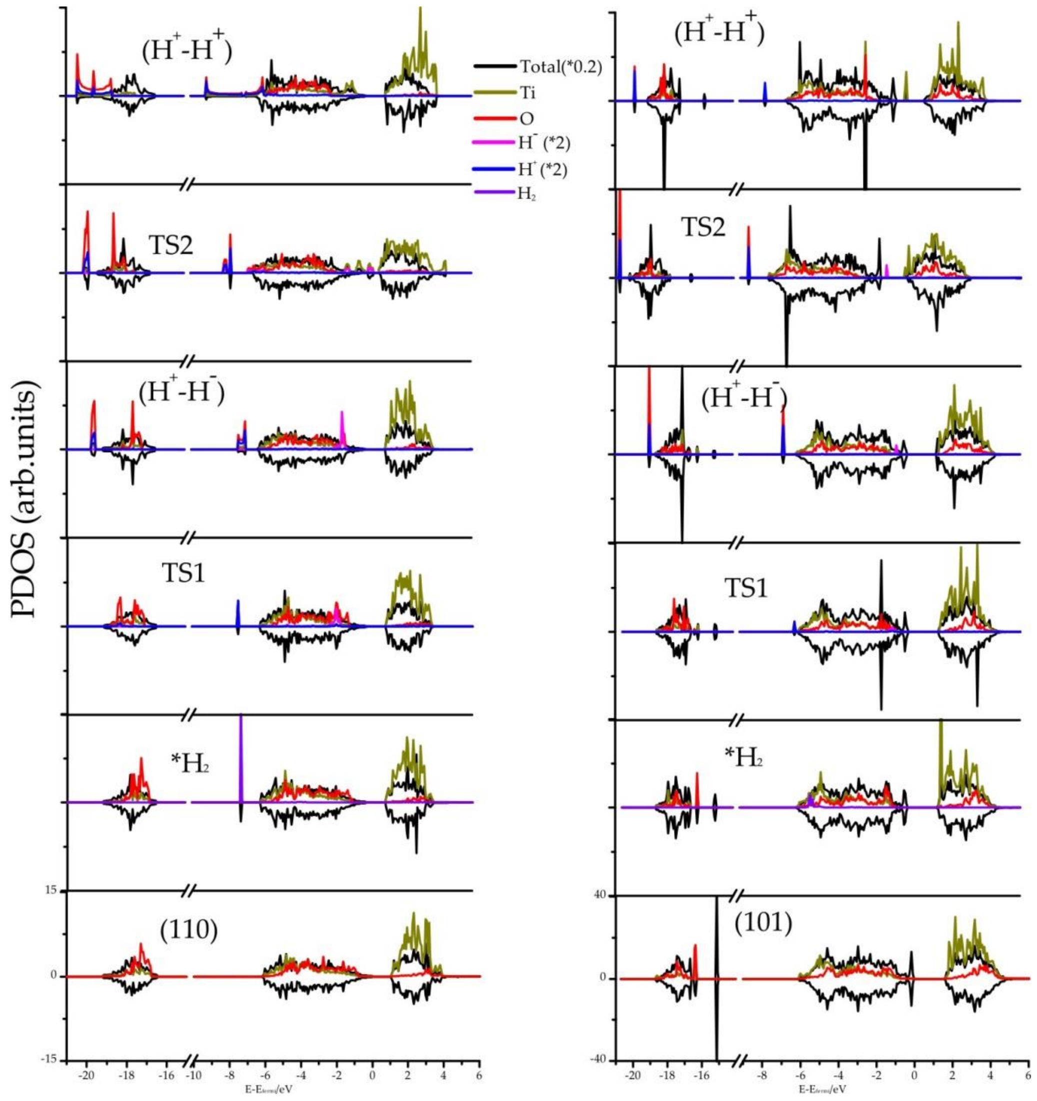

Figure S4. Total and Projected Density of State (PDOS) of the TiO_2 slab, $*H_2$, TS, and $*(H^+, H^-)$, $*(H^+-H^+)$ for the (110) (left) and (101) (right) surfaces. For the PDOS, only the Ti, O involved in the two processes are projected. Positive DOS correspond to spin up, negative to spin down.

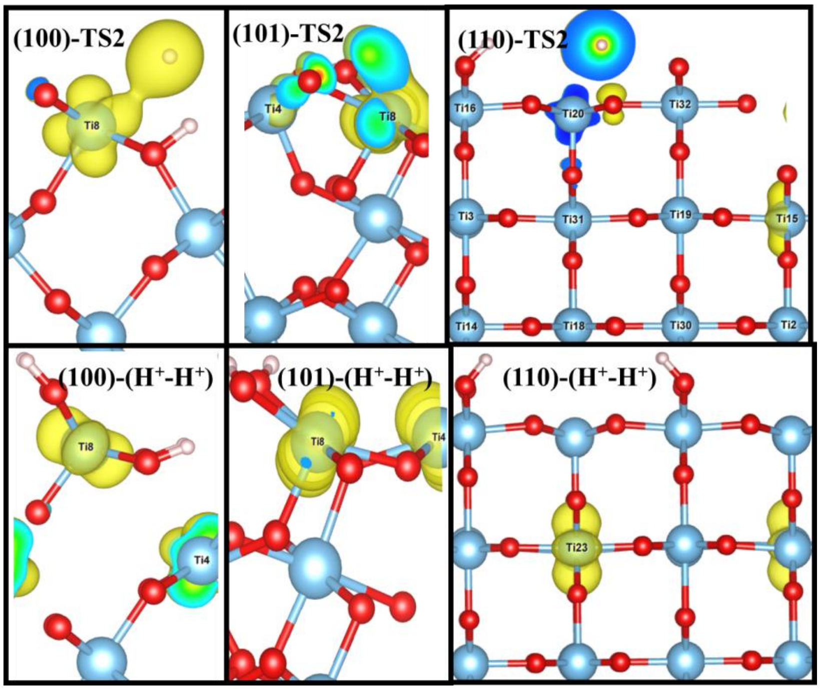

Figure S5. Spin Density of TS 2 and (H^+-H^+) species indicating the distribution of unpaired electrons on rutile TiO_2 (100), (101) and (110) facets.

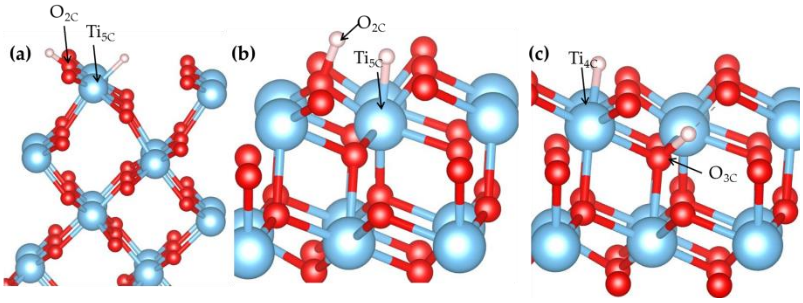

Figure S6. Structures of (H^+-H^-) for the other adsorption modes considered on (100) facet (a) and (101) facet(b,c).

Tests on IR frequency calculations

Table 1. Computed IR frequencies for (110) TiO_2-(H^+, H^-) species as a function of the functional, cut-off, U test and with dipole corrections.

system	test	method	υ(OH)/cm^{-1}	υ(Ti-H)/cm^{-1}
(110)	functional	PBE+U(4eV)-400eV	3606.5	1653.8
		LDA+U(4eV)-400eV	3607.8	1638.9
		PW91+U(4eV)-400eV	3608.1	1648.7
		PBE-Sol+U(4eV)-400eV	3631.8	1665.0
	cut-off test	300 eV	3486.2	1644.1
		400 eV	3606.5	1653.87
		500 eV	3610.9	1652.3
		600 eV	3603.0	1642.9
		700 eV	3612.8	1655.8
	choice of U	U=3eV	3605.6	1658.4
		U=4eV	3606.5	1653.8
		U=5eV	3606.2	1640.3
		U=6eV	3607.6	1630.6
		U=7eV	3609.4	1609.0
	with dipole corrections	PBE+U(4eV)-400eV	3604.8	1610.3

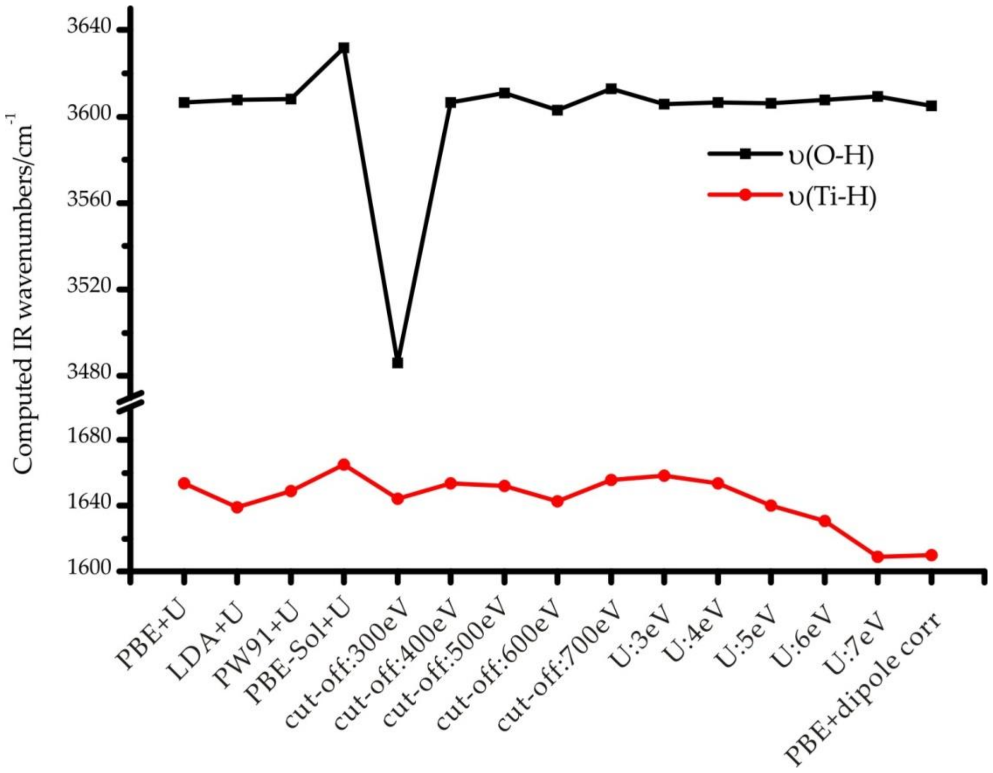

Figure S7. Computed IR vibrations of Ti-H and O-H in (H^+, H^-) species for the (110) termination as a function of the functional, plane wave cut-off, choice of U, and with dipole corrections.

Table S2. Computed IR frequencies for (100) TiO_2-(H^+, H^-) species as a function of the functional, cut-off, U test and with dipole corrections.

system	test	method	υ(OH)	υ(Ti-H)
(100)	functional	PBE+U(4eV)-400eV	2976.5	1768.7
		LDA+U(4eV)-400eV	2954.5	1718.6
		PW91+U(4eV)-400eV	2956.1	1724.3
		PBE-Sol+U(4eV)-400eV	2990.0	1736.2
	cut-off	300 eV	2810.3	1721.3
		400 eV	2976.5	1768.7
		500 eV	2955.8	1726.9
		600 eV	2957.8	1723.8
		700 eV	2949.0	1714.6
	choice of U	U=3eV	2958.6	1732.2
		U=4eV	2976.5	1768.7
		U=5eV	2949.6	1719.0
		U=6eV	2945.1	1710.3
		U=7eV	2940.9	1699.4
	with dipole corrections	PBE+U(4eV)-400eV	2945.0	1726.1

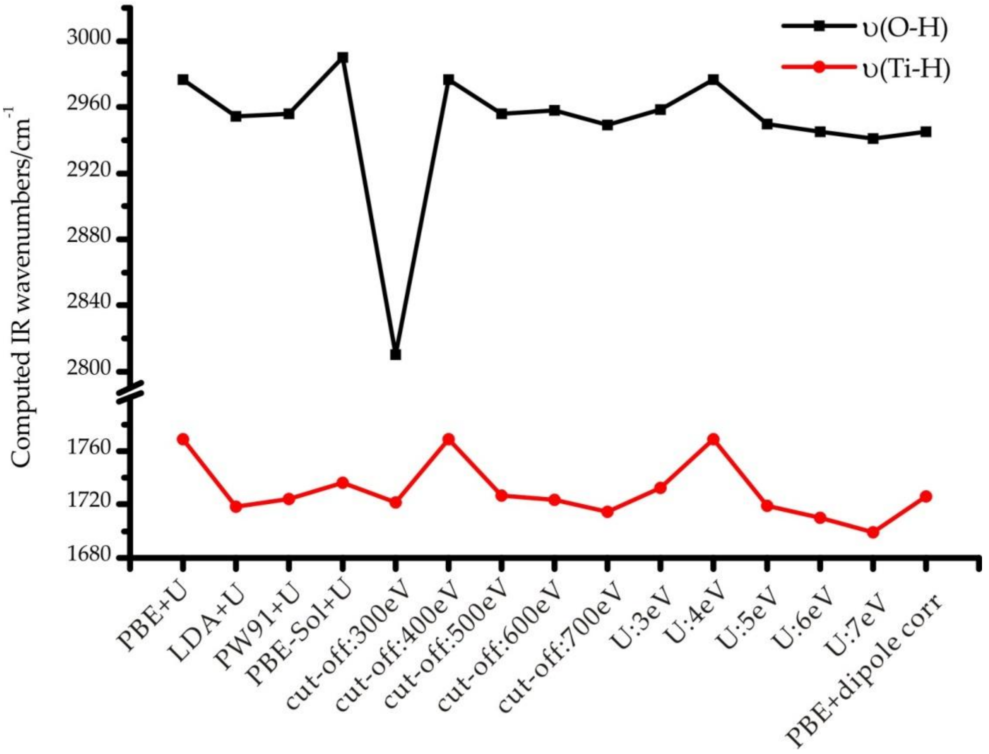

Figure S8. Computed IR vibrations of Ti-H and O-H in (H^+, H^-) species for the (100) termination as a function of the functional, plane wave cut-off, choice of U, and with dipole corrections..

Table S3. Computed IR frequencies for (001) TiO_2-(H^+, H^-) species as a function of the functional, cut-off, U test and with dipole corrections.

system	test	method	υ(OH)	υ(Ti-H)
(001)	functional	PBE+U(4eV)-400eV	3742.9	1644.8
		LDA+U(4eV)-400eV	3745.9	1649.3
		PW91	3746.8	1653.8
	cut-off	300 eV	3628.3	1646.6
		400 eV	3742.9	1644.8
		500 eV	3743.1	1645.2
		600 eV	3750.2	1651.3
		700 eV	3745.0	1641.8
	choice of U	U=3eV	3743.9	1648.2
		U=4eV	3742.9	1644.8
		U=5eV	3744.0	1656.8
		U=6eV	3744.0	1660.5
		U=7eV	3744.2	1663.7
	with dipole corrections	PBE+U(4eV)-400eV	3728.71	1623.37

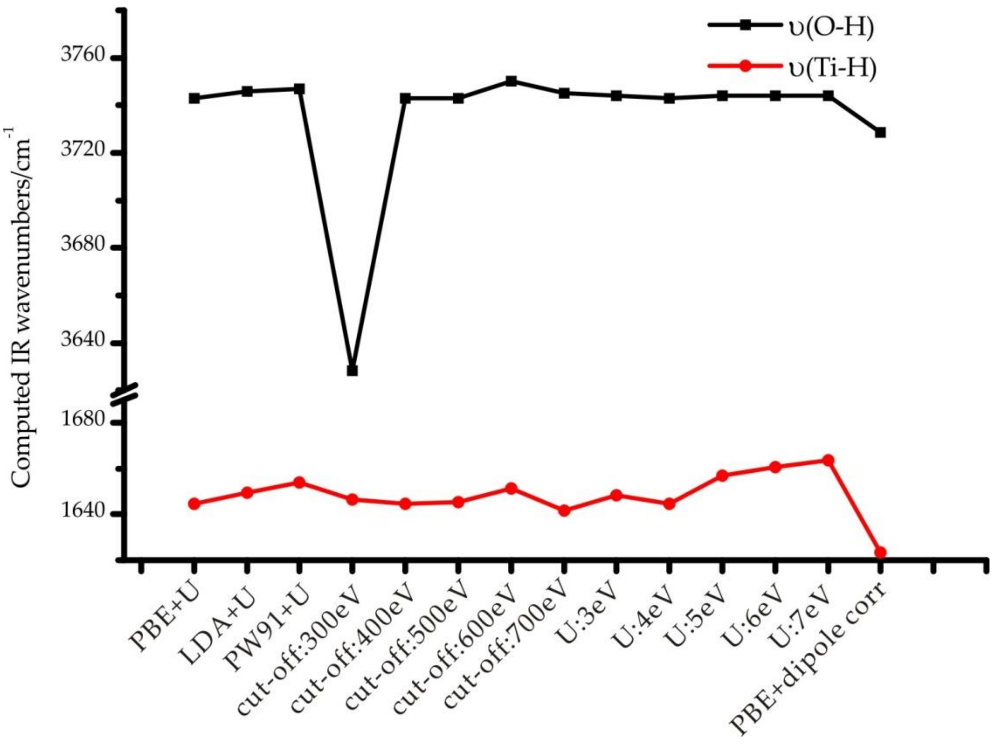

Figure S9. Computed IR vibrations of Ti-H and O-H in (H^+, H^-) specie for the (001) termination as a function of the functional, plane wave cut-off, choice of U, and with dipole corrections.

Table S4. Computed IR frequencies for (101) TiO_2-(H^+, H^-) species as a function of the functional, cut-off, U test and with dipole corrections.

system	test	method	υ(OH)	υ(Ti-H)
(101)	functional	PBE+U(4eV)-400eV	3622.4	1577.5
		LDA+U(4eV)-400eV	3618.3	1581.2
		PW91	3620.2	1586.1
	cut-off	300 eV	3496.0	1585.1
		400 eV	3622.4	1577.5
		500 eV	3623.9	1584.0
		600 eV	3625.4	1580.0
		700 eV	3619.2	1570.2
	choice of U	U=3eV	3617.5	1589.8
		U=4eV	3622.4	1577.5
		U=5eV	3617.9	1586.0
		U=6eV	3618.1	1582.3
		U=7eV	3618.2	1577.5
	with dipole corrections	PBE+U(4eV)-400eV	3615.5	1566.5

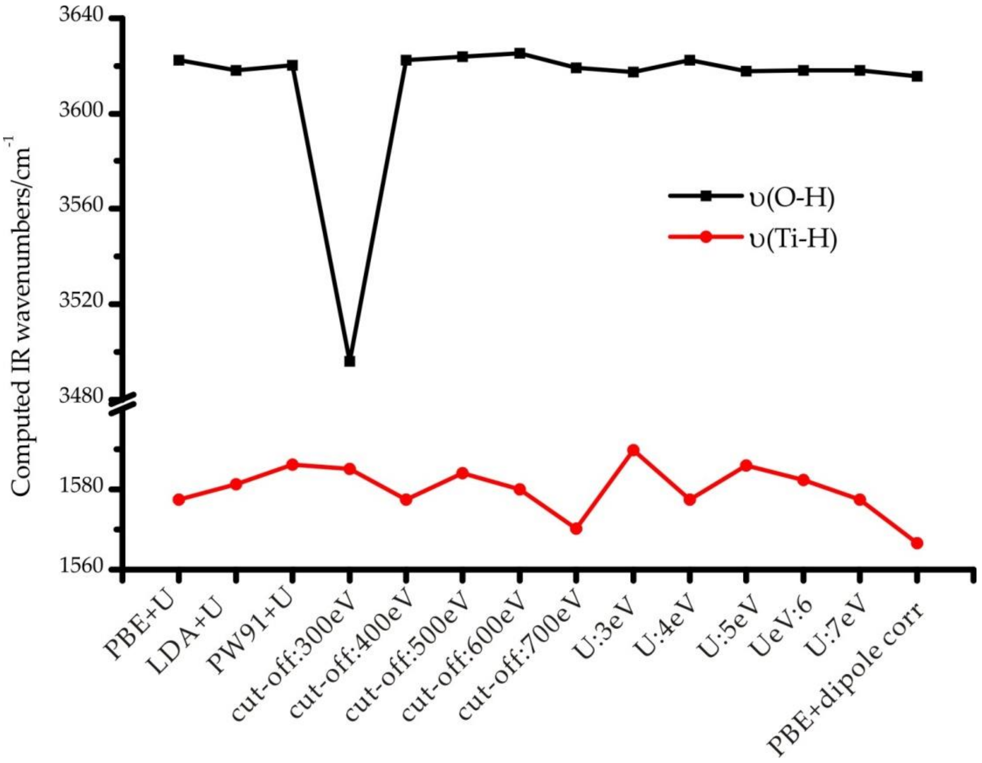

Figure S10. Computed IR vibrations of Ti-H and O-H in (H^+, H^-) specie for the (101) termination as a function of the functional, plane wave cut-off, choice of U, and with dipole corrections.

Table 5. Selected previous studies of experimental and calculated stretching frequencies of various OH in TiO_2 systems, and Ti-H supported on silica.

	System	Method	υ(OH)/cm^{-1}	υ(Ti-H)/cm^{-1}
Experimental data[1-6]	TiO_2(110) single crystal	HREELS	3690	
	TiO_2(110)	IRAS	3711	
	rutile (110)	Infrared spectroscopic techniques	3410 and 3650	

	rutile (100)	Infrared spectroscopic techniques	3550	
	rutile (101)	Infrared spectroscopic techniques	3680 and 3610	
	rutile (110)	Infrared spectroscopic techniques	3655 and 3410	
	Silica-Supported Titanium Hydrides	Infrared spectroscopic techniques		1706, 1692, 1679, and 1647 cm^{-1}
	anatase TiO_2	FT-IR	3640-3735	
Theoretical data[7,8]	rutile (110)	The vibrational frequencies were calculated numerically	3700	
	anatase (001): Ti_V-μ1OH	finite difference approach +anharmonicity corrections (71–73 cm^{-1})	3760(3746–3751)	
	anatase (110): Ti_V- μ1OH		3728	
	anatase (100): Ti_{VI}-μ1OH		3688	

Table S6. Comparison of computed IR wavenumbers (cm^{-1}) of Ti–H and O–H Stretching Modes of (H^+,H^-) Species for the four terminations studied with and without grimme D3 corrections using Finite Differences (FD) method.

	**υ(Ti-H)/cm^{-1}**		υ(OH)/cm^{-1}	
**Slab**	**FD-PBE-U**	FD-PBE-U-D3	FD-PBE-U	FD-PBE-U-D3
(001)	1656.94	1656.47	3763.85	3766.78
(100)	1726.98	1718.94	2959.92	2958.67
(110)	1654.55	1659.93	3618.55	3623.73
(101)	1570.11	1583.89	3626.45	3625.28

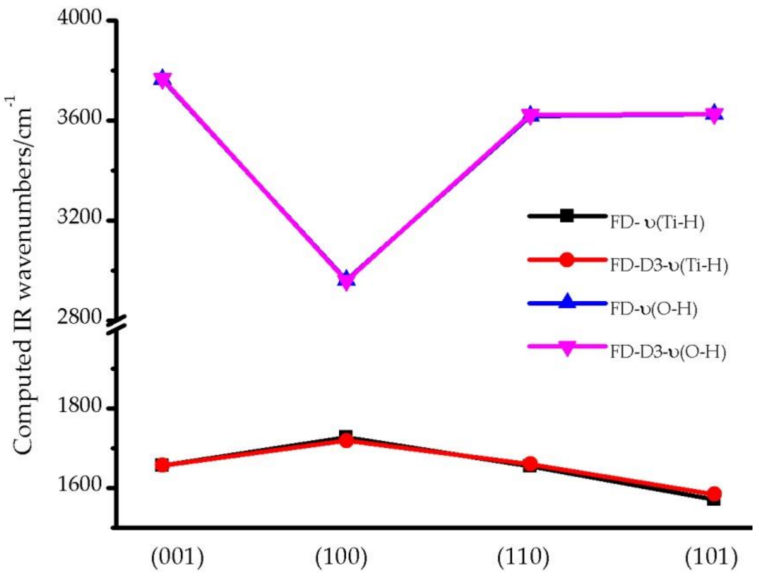

Figure S11. Comparison of computed IR wavenumbers (cm^{-1}) of Ti–H and O–H Stretching Modes of (H^+,H^-) Species for the four terminations studied with and without grimme D3 corrections using Finite Differences (FD) method.

Grimme D3 dispersion correction

Table 7. Relative energy (eV) of adsorption of molecular hydrogen on four surfaces and subsequent heterolytic dissociation generating (H^{+}-H^{-}) species (in parentheses, the D3 corrected values). ΔE(eV) is the reaction energy for heterolytic dissociation of adsorbed hydrogen ΔE=E(H^{+}-H^{-})-E(*H_2). The reference is taken as the sum of dihydrogen and the bare slab.

	**(001)**	**(100)**	**(110)**	**(101)**
slab+H_2	0.00(0.00)	0.00(0.00)	0.00(0.00)	0.00(0.00)
*H_2	-0.13(-0.21)	-0.08(-0.23)	0.10(-0.24)	-0.14(-0.25)
TS1	0.43(0.32)	1.00(0.62)	0.60(0.29)	0.65(0.53)
$E^{\neq}$	0.56(0.53)	1.08(0.85)	0.50(0.54)	0.79(0.79)
(H^{+}-H^{-})	-0.21(-0.36)	0.60(0.38)	0.22(-0.18)	-0.22(-0.35)
ΔE	-0.08(-0.15)	0.68(0.61)	0.12(0.06)	-0.08(-0.10)

Figure S12. The energy profile of the adsorption of molecular hydrogen on four surfaces and subsequent heterolytic dissociation generating (H^{+}-H^{-}) species.(a) without D3 correction; (b) with D3 correction.

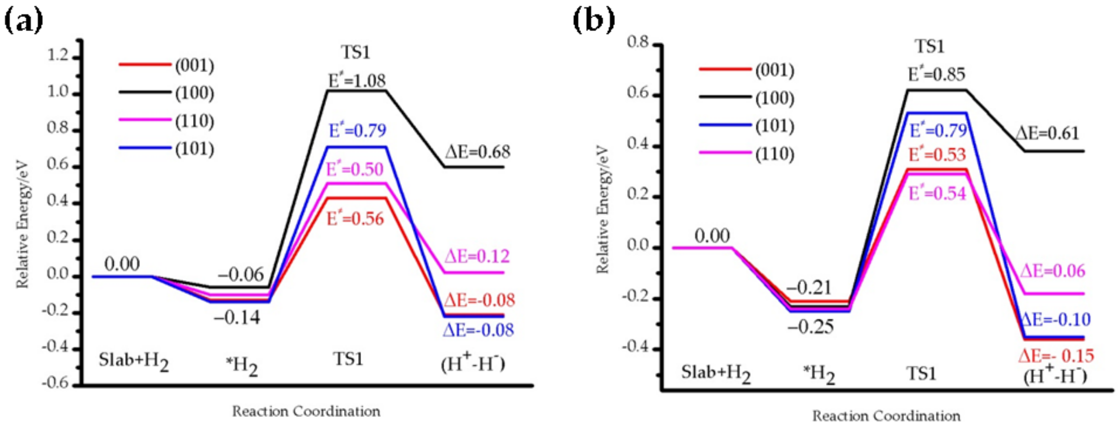

Figure S12. The energy profile of the adsorption of molecular hydrogen on four surfaces and subsequent heterolytic dissociation generating (H^{+}-H^{-}) species.(a) without D3 correction; (b) with D3 correction.

References:

1. Primet, M.; Pichat, P.; Mathieu, M.V. Infrared study of the surface of titanium dioxides. I. Hydroxyl groups. *The Journal of Physical Chemistry* **1971**, *75*, 1216-1220, doi: org/10.1021/j100679a007
2. Jones, P.; Hockey, J. Infra-red studies of rutile surfaces. Part 2.—hydroxylation, hydration and structure of rutile surfaces. *Transactions of the Faraday Society* **1971**, *67*, 2679-2685. doi:10.1039/TF9716702679
3. M. A. Henderson., An HREELS and TPD study of water on TiO_2(110): the extent of molecular versus dissociative adsorption. *Surf*. *Sci*. **1996**,355, 151-166, doi: org/10.1016/0039-6028(95)01357-1
4. M. A. Henderson., W. S. Epling., C. H. F. Peden., C. L. Perkins. Insights into photoexcited electron scavenging processes on TiO_2 obtained from studies of the reaction of O_2 with OH groups

adsorbed at electronic defects on TiO_2(110). *J. Phys. Chem. B.* **2003**, 107, 534-545, doi: org/10.1021/jp0262113
5. N. G. Petrik., G. A. Kimmel. Reaction kinetics of water molecules with oxygen vacancies on rutile TiO_2(110). *J. Phys. Chem. C.* **2015**, 119, 23059-23067, doi: org/10.1021/acs.jpcc.5b07526
6. Pan, J.M.; Maschhoff, B.L.; Diebold, U.; Madey, T.E. Interaction of water, oxigen, and hydrogen with TiO2 surfaces having different defect densities. *J. Vac. Sci. Technol. A-Vac. Surf. Films* **1992**, *10*, 2470-2476, doi:10.1116/1.577986.
7. C. Arrouvel, M. Digne, M. Breysse, H. Toulhoat and P. Raybaud, Effects of morphology on surface hydroxyl concentration: a DFT comparison of anatase–TiO_2 and γ-alumina catalytic supports. *J. Catal.* **2004**, 222 ,1, 152-166, doi: org/10.1016/j.jcat.2003.10.016
8. Buchholz, M., Wöll, C., Interaction of carboxylic acids with rutile TiO_2(110): IR-investigations of terephthalic and benzoic acid adsorbed on a single crystal substrate, *Surf. Sci.* **2016**, 643, 117–123. doi: org/10.1016/j.susc.2015.08.006

Structures (POSCAR format)

A. Surfaces

※ (002)-bare

```
      1.00000000000000
      4.6612049999999998    0.0000000000000000    0.0000000000000000
      0.0000000000000000    4.6612049999999998    0.0000000000000000
      0.0000000000000000    0.0000000000000000   30.3668990000000001
  Ti   O
      8    16
Selective dynamics
Direct
   0.0000000000000000  0.1955000000000027  0.0000000000000000   F   F   F
   0.0000000000000000  0.1955000000000027  0.0975400000000022   F   F   F
  -0.0000000000000000  0.1955000000000027  0.1993034740869846   T   T   T
  -0.0000000000000000  0.1955000000000027  0.3050444256455350   T   T   T
   0.5000000000000000  0.6955000100000035  0.0487699999999975   F   F   F
   0.5000000000000000  0.6955000100000035  0.1463100100000005   F   F   F
   0.5000000000000000  0.6955000100000035  0.2442446311049732   T   T   T
   0.5000000000000000  0.6955000100000035  0.3402608140993794   T   T   T
   0.3045000199999990  0.5000000000000000  0.0000000000000000   F   F   F
   0.3045000199999990  0.5000000000000000  0.0975400000000022   F   F   F
   0.3040923561607641  0.4995923361607651  0.1969375971180245   T   T   T
   0.3020707423384540  0.4975707223384550  0.2962941918885365   T   T   T
   0.6955000100000035  0.8909999700000029  0.0000000000000000   F   F   F
   0.6955000100000035  0.8909999700000029  0.0975400000000022   F   F   F
   0.6959076738392383  0.8914076338392377  0.1969375971180245   T   T   T
   0.6979292876615486  0.8934292476615480  0.2962941918885365   T   T   T
   0.1955000000000027  0.0000000000000000  0.0487699999999975   F   F   F
   0.1955000000000027  0.0000000000000000  0.1463100100000005   F   F   F
   0.1948365212177177  0.0006634787822848  0.2486357067529323   T   T   T
   0.2137838403715509  0.9817161596284518  0.3493237249367281   T   T   T
   0.8044999899999965  0.3909999899999974  0.0487699999999975   F   F   F
   0.8044999899999965  0.3909999899999974  0.1463100100000005   F   F   F
   0.8051634687822815  0.3903365112177123  0.2486357067529323   T   T   T
   0.7862161496284483  0.4092838303715457  0.3493237249367281   T   T   T
```

※ (002)-H_2

```
       1.00000000000000
      4.6612049999999998    0.0000000000000000    0.0000000000000000
      0.0000000000000000    4.6612049999999998    0.0000000000000000
```

0.0000000000000000 0.0000000000000000 30.3668990000000001 Ti O
H
8 16 2
Selective dynamics
Direct

0.0000000000000000	0.1955000000000027	0.0000000000000000	F	F	F
0.0000000000000000	0.1955000000000027	0.0975400000000022	F	F	F
0.0003322181453851	0.1952251771420842	0.1992878843111771	T	T	T
0.0023618700960367	0.1934788647532495	0.3047715833142486	T	T	T
0.5000000000000000	0.6955000100000035	0.0487699999999975	F	F	F
0.5000000000000000	0.6955000100000035	0.1463100100000005	F	F	F
0.5010510033829616	0.6945778684424891	0.2443501170867095	T	T	T
0.5024519422906005	0.6935884689865255	0.3405437510968077	T	T	T
0.3045000199999990	0.5000000000000000	0.0000000000000000	F	F	F
0.3045000199999990	0.5000000000000000	0.0975400000000022	F	F	F
0.3046864844076886	0.4991051930609143	0.1969513957978715	T	T	T
0.3039405875662026	0.4963290099968189	0.2964845811395622	T	T	T
0.6955000100000035	0.8909999700000029	0.0000000000000000	F	F	F
0.6955000100000035	0.8909999700000029	0.0975400000000022	F	F	F
0.6966260691738655	0.8907201819006042	0.1969443571982689	T	T	T
0.6997137132910302	0.8916268708053822	0.2964621670420085	T	T	T
0.1955000000000027	0.0000000000000000	0.0487699999999975	F	F	F
0.1955000000000027	0.0000000000000000	0.1463100100000005	F	F	F
0.1962549237697085	0.9996060407803183	0.2485239838190635	T	T	T
0.2148942567028567	0.9811182023017889	0.3490882051861650	T	T	T
0.8044999899999965	0.3909999899999974	0.0487699999999975	F	F	F
0.8044999899999965	0.3909999899999974	0.1463100100000005	F	F	F
0.8059742523805212	0.3894905624392745	0.2485267443639959	T	T	T
0.7896563394584089	0.4059631840493360	0.3490670737666857	T	T	T
0.4549076049793557	0.7487472082398272	0.4181494495439089	T	T	T
0.5705062529753432	0.6331133267179951	0.4174521164292490	T	T	T

※ (002)-H^+-H^-

1.00000000000000
4.6612049999999998 0.0000000000000000 0.0000000000000000
0.0000000000000000 4.6612049999999998 0.0000000000000000
0.0000000000000000 0.0000000000000000 30.3668990000000001
Ti O H
8 16 2
Selective dynamics
Direct

0.0000000000000000	0.1955000000000027	0.0000000000000000	F	F	F
0.0000000000000000	0.1955000000000027	0.0975400000000022	F	F	F

-0.0007968484301485	0.1962968484301512	0.1977815760296955	T	T	T
0.0074643400115428	0.1880356599884599	0.2997006543040509	T	T	T
0.5000000000000000	0.6955000100000035	0.0487699999999975	F	F	F
0.5000000000000000	0.6955000100000035	0.1463100100000005	F	F	F
0.5034973219974450	0.6920026880025585	0.2459735280226975	T	T	T
0.4762305095529218	0.7192695004470819	0.3442192095641228	T	T	T
0.3045000199999990	0.5000000000000000	0.0000000000000000	F	F	F
0.3045000199999990	0.5000000000000000	0.0975400000000022	F	F	F
0.3044069661236521	0.5001224425142579	0.1970255797018548	T	T	T
0.3002801112292930	0.4991340067413219	0.2969667668436415	T	T	T
0.6955000100000035	0.8909999700000029	0.0000000000000000	F	F	F
0.6955000100000035	0.8909999700000029	0.0975400000000022	F	F	F
0.6953775674857459	0.8910930238763496	0.1970255797018548	T	T	T
0.6963660032586746	0.8952198787707016	0.2969667668436415	T	T	T
0.1955000000000027	0.0000000000000000	0.0487699999999975	F	F	F
0.1955000000000027	0.0000000000000000	0.1463100100000005	F	F	F
0.1949887648370353	0.0005112351629603	0.2476175901205793	T	T	T
0.1919120687838471	0.0035879312161485	0.3476074538817706	T	T	T
0.8044999899999965	0.3909999899999974	0.0487699999999975	F	F	F
0.8044999899999965	0.3909999899999974	0.1463100100000005	F	F	F
0.8049859172854880	0.3905140627145131	0.2475342853793073	T	T	T
0.7880202268809268	0.4074797531190744	0.3488777128960971	T	T	T
0.5004885808027651	0.6950114391972321	0.4012992240930531	T	T	T
0.8481420228767608	0.3473579671232412	0.3781473132712567	T	T	T

※(002)-TS1

1.00000000000000
4.6612049999999998 0.0000000000000000 0.0000000000000000
0.0000000000000000 4.6612049999999998 0.0000000000000000
0.0000000000000000 0.0000000000000000 30.3668990000000001
Ti O H
8 16 2
Selective dynamics
Direct

0.0000000000000000	0.1955000000000027	0.0000000000000000	F	F	F
0.0000000000000000	0.1955000000000027	0.0975400000000022	F	F	F
0.0029315047703022	0.1923962380738615	0.1987680999377446	T	T	T
0.0129490893627846	0.1815129450191506	0.3028467571756508	T	T	T
0.5000000000000000	0.6955000100000035	0.0487699999999975	F	F	F
0.5000000000000000	0.6955000100000035	0.1463100100000005	F	F	F
0.5083464289270054	0.6868500021500026	0.2454658464781900	T	T	T
0.4968415925365832	0.6969536835015404	0.3432798047643502	T	T	T
0.3045000199999990	0.5000000000000000	0.0000000000000000	F	F	F

0.3045000199999990	0.5000000000000000	0.0975400000000022	F	F	F
0.3080515012668670	0.4961412396507541	0.1971978156690213	T	T	T
0.3120245992706927	0.4875123126502956	0.2972783294904460	T	T	T
0.6955000100000035	0.8909999700000029	0.0000000000000000	F	F	F
0.6955000100000035	0.8909999700000029	0.0975400000000022	F	F	F
0.6993556229011375	0.8870331730338208	0.1972123040480867	T	T	T
0.7071128583042068	0.8827008320723522	0.2973504825444166	T	T	T
0.1955000000000027	0.0000000000000000	0.0487699999999975	F	F	F
0.1955000000000027	0.0000000000000000	0.1463100100000005	F	F	F
0.2006493968332705	0.9944586296223648	0.2484949492699826	T	T	T
0.2149698336780546	0.9801454310131632	0.3477866526819398	T	T	T
0.8044999899999965	0.3909999899999974	0.0487699999999975	F	F	F
0.8044999899999965	0.3909999899999974	0.1463100100000005	F	F	F
0.8111794933839889	0.3836149945824445	0.2484943492055720	T	T	T
0.8028071329038369	0.3930010702668199	0.3508866220536788	T	T	T
0.5755420711312880	0.6146375082375641	0.4043939959586424	T	T	T
0.7307048329368229	0.4620423458252034	0.3850134762888047	T	T	T

※(002)-TS2

Ti O H
1.00000000000000
4.6612049999999998 0.0000000000000000 0.0000000000000000
0.0000000000000000 4.6612049999999998 0.0000000000000000
0.0000000000000000 0.0000000000000000 30.3668990000000001
Ti O H
8 16 2
Selective dynamics
Direct

0.0000000000000000	0.1955000000000027	0.0000000000000000	F	F	F
0.0000000000000000	0.1955000000000027	0.0975400000000022	F	F	F
0.0014144947563292	0.1942695914033768	0.1969018884841363	T	T	T
0.0131419094772985	0.1838458462935561	0.2991890948084995	T	T	T
0.5000000000000000	0.6955000100000035	0.0487699999999975	F	F	F
0.5000000000000000	0.6955000100000035	0.1463100100000005	F	F	F
0.5078118400777977	0.6879438836518873	0.2465143813797936	T	T	T
0.4997077692445395	0.7015386969253092	0.3440972050684749	T	T	T
0.3045000199999990	0.5000000000000000	0.0000000000000000	F	F	F
0.3045000199999990	0.5000000000000000	0.0975400000000022	F	F	F
0.3027676998291667	0.4924805896389756	0.1957132738644561	T	T	T
0.3087327341576238	0.4823672657997402	0.3018454625371225	T	T	T
0.6955000100000035	0.8909999700000029	0.0000000000000000	F	F	F
0.6955000100000035	0.8909999700000029	0.0975400000000022	F	F	F
0.7023974472116524	0.8937524593196613	0.1957226972832722	T	T	T

0.7110907121797380	0.8940060786288412	0.3010656090988665	T	T	T
0.1955000000000027	0.0000000000000000	0.0487699999999975	F	F	F
0.1955000000000027	0.0000000000000000	0.1463100100000005	F	F	F
0.1977546628673606	0.9972353125704696	0.2501518904465682	T	T	T
0.2124229399124736	0.9824116327712601	0.3543991688411517	T	T	T
0.8044999899999965	0.3909999899999974	0.0487699999999975	F	F	F
0.8044999899999965	0.3909999899999974	0.1463100100000005	F	F	F
0.8177487190049216	0.3820948882413980	0.2509380156491487	T	T	T
0.7980172509590243	0.3966353037439359	0.3555235080921110	T	T	T
0.3422298880560332	0.8431005951391592	0.3973964980107870	T	T	T
0.9102695681205897	0.3476295934271332	0.3814356058955297	T	T	T

※ (002)-H^+-H^+

1.00000000000000

4.6612049999999998	0.0000000000000000	0.0000000000000000
0.0000000000000000	4.6612049999999998	0.0000000000000000
0.0000000000000000	0.0000000000000000	30.3668990000000001

Ti O H

8 16 2

Selective dynamics

Direct

0.0000000000000000	0.1955000000000027	0.0000000000000000	F	F	F
0.0000000000000000	0.1955000000000027	0.0975400000000022	F	F	F
0.0008991255129241	0.1941854902610489	0.1955384765382058	T	T	T
0.9841096108057232	0.2177716849690477	0.2953473048046902	T	T	T
0.5000000000000000	0.6955000100000035	0.0487699999999975	F	F	F
0.5000000000000000	0.6955000100000035	0.1463100100000005	F	F	F
0.4901023615959322	0.7047154981339698	0.2487783966066029	T	T	T
0.5452948608506497	0.6575641937988884	0.3513917446843169	T	T	T
0.3045000199999990	0.5000000000000000	0.0000000000000000	F	F	F
0.3045000199999990	0.5000000000000000	0.0975400000000022	F	F	F
0.2974552157874973	0.4976191530653293	0.1957285401376275	T	T	T
0.2993419694637933	0.4791965148217639	0.3052342266230700	T	T	T
0.6955000100000035	0.8909999700000029	0.0000000000000000	F	F	F
0.6955000100000035	0.8909999700000029	0.0975400000000022	F	F	F
0.6974884826882275	0.8984278820719411	0.1957367419946908	T	T	T
0.7133910673243825	0.8999812949247767	0.3060068617729105	T	T	T
0.1955000000000027	0.0000000000000000	0.0487699999999975	F	F	F
0.1955000000000027	0.0000000000000000	0.1463100100000005	F	F	F
0.1837970380593047	0.0094269417779553	0.2504856047695752	T	T	T
0.2682007344631050	0.6710994920279780	0.4006332555110219	T	T	T
0.8044999899999965	0.3909999899999974	0.0487699999999975	F	F	F
0.8044999899999965	0.3909999899999974	0.1463100100000005	F	F	F

0.8011299692717132	0.3963460315465338	0.2484721548243076	T	T	T
0.8526190593269185	0.3466495310596264	0.3598800119923815	T	T	T
0.2247208243921673	0.8258368972598110	0.4209339939032165	T	T	T
0.0060085779327261	0.4438878718643945	0.3773978289724536	T	T	T

※(100)-bare

1.00000000000000

4.6612049999999998	0.0000000000000000	0.0000000000000000
0.0000000000000000	2.9619610000000001	0.0000000000000000
0.0000000000000000	0.0000000000000000	37.4114300000000028

Ti O

8 16

Selective dynamics

Direct

0.9907484399999973	0.0000000000000000	0.0225501800000032	F	F	F
0.9933728500000001	0.0000000000000000	0.1430838499999965	F	F	F
0.0012269971612176	0.0000000000000000	0.2647141009436588	T	T	T
-0.0018283757240420	0.0000000000000000	0.3928539551519645	T	T	T
0.4921791399999975	0.5000000000000000	0.0828389200000004	F	F	F
0.4956807800000007	0.5000000000000000	0.2028307400000031	F	F	F
0.4995269288052293	0.5000000000000000	0.3285231949641199	T	T	T
0.4901137428157775	0.5000000000000000	0.4552154979437046	T	T	T
0.6989695600000019	0.0000000000000000	0.0618577799999969	F	F	F
0.6879715600000011	0.0000000000000000	0.1802123200000025	F	F	F
0.7045681285534378	0.0000000000000000	0.3043387373218517	T	T	T
0.7400515282680256	0.0000000000000000	0.4341148286857447	T	T	T
0.2021347599999999	0.5000000000000000	0.0441403499999993	F	F	F
0.1872477100000012	0.5000000000000000	0.1657281499999996	F	F	F
0.2037295507440408	0.5000000000000000	0.2889997247748871	T	T	T
0.2189177591657117	0.5000000000000000	0.4159365337338155	T	T	T
0.2957171099999982	0.0000000000000000	0.1053988299999986	F	F	F
0.3054150199999981	0.0000000000000000	0.2256907800000008	F	F	F
0.2994912162793038	0.0000000000000000	0.3525671043798094	T	T	T
0.3636547152586171	0.0000000000000000	0.4824267100281232	T	T	T
0.8587117053733961	0.5000000000000000	0.9949993661784378	T	T	T
0.7982593600000030	0.5000000000000000	0.1207107099999973	F	F	F
0.8054150199999981	0.5000000000000000	0.2397132700000029	F	F	F
0.7980335084283420	0.5000000000000000	0.3689380857514415	T	T	T

※(100)-H_2

1.00000000000000000000

4.6612049999999998	0.0000000000000000	0.0000000000000000

0.0000000000000000 2.9619610000000001 0.0000000000000000
0.0000000000000000 0.0000000000000000 37.4114300000000028
Ti O H
8 16 2
Selective dynamics
Direct

0.9907499999999985	0.0000000000000000	0.0225500000000025	F	F	F
0.9933699899999979	0.0000000000000000	0.1430799899999968	F	F	F
0.0011511493819829	0.9997153200669053	0.2647869129927501	T	T	T
-0.0014265615683368	0.9981281881137597	0.3930085992793760	T	T	T
0.4921799899999968	0.5000000000000000	0.0828399999999974	F	F	F
0.4956800100000009	0.5000000000000000	0.2028299999999987	F	F	F
0.4996069148683389	0.4987067722457575	0.3286485838782707	T	T	T
0.4901062517385200	0.4970146591202269	0.4555935839960930	T	T	T
0.6989700000000028	0.0000000000000000	0.0618600000000029	F	F	F
0.6879699699999975	0.0000000000000000	0.1802099900000016	F	F	F
0.7039735738810442	0.9989428063694372	0.3043548202183810	T	T	T
0.7350952976424762	0.9974703056654657	0.4341070940504281	T	T	T
0.2021300099999976	0.5000000000000000	0.0441399999999987	F	F	F
0.1872499999999988	0.5000000000000000	0.1657300099999972	F	F	F
0.2030984750866620	0.4995572868863244	0.2890965087855573	T	T	T
0.2173234647253838	0.4979193825196236	0.4161306338403511	T	T	T
0.2957200099999966	0.0000000000000000	0.1054000099999968	F	F	F
0.3054200199999997	0.0000000000000000	0.2256900100000010	F	F	F
0.2999582358232483	0.9986237744110973	0.3527968686578333	T	T	T
0.3468667978206949	0.9969864696525212	0.4818350674703918	T	T	T
0.8585603044367173	0.4999872570055785	0.9949570312406069	T	T	T
0.7982599899999983	0.5000000000000000	0.1207100000000025	F	F	F
0.8054199700000026	0.5000000000000000	0.2397100100000031	F	F	F
0.7990343289414876	0.4982302764667723	0.3689261578577177	T	T	T
0.7765188257365757	0.5414039214344972	0.5089706755750172	T	T	T
0.8992154533541895	0.4308940201813546	0.4988714975453386	T	T	T

※(100)-TS1

1.00000000000000
4.6612049999999998 0.0000000000000000 0.0000000000000000
0.0000000000000000 2.9619610000000001 0.0000000000000000
0.0000000000000000 0.0000000000000000 37.4114300000000028
Ti O H
8 16 2
Selective dynamics
Direct

0.9907499999999985	0.0000000000000000	0.0225500000000025	F	F	F

0.9933699899999979	0.0000000000000000	0.1430799899999968	F	F	F
0.0018038990704596	0.0002620724453308	0.2643382250692738	T	T	T
0.0016205539087153	0.0017548325033800	0.3909208291919626	T	T	T
0.4921799899999968	0.5000000000000000	0.0828399999999974	F	F	F
0.4956800100000009	0.5000000000000000	0.2028299999999987	F	F	F
0.4998615424596764	0.5018755811449154	0.3275669937626944	T	T	T
0.4620918208947060	0.5151408140896336	0.4579377846614179	T	T	T
0.6989700000000028	0.0000000000000000	0.0618600000000029	F	F	F
0.6879699699999975	0.0000000000000000	0.1802099900000016	F	F	F
0.7037982023940472	0.0012943396288634	0.3036130107132777	T	T	T
0.7328805229582045	0.9991352036723171	0.4341170729260853	T	T	T
0.2021300099999976	0.5000000000000000	0.0441399999999987	F	F	F
0.1872499999999988	0.5000000000000000	0.1657300099999972	F	F	F
0.2041953209258267	0.5005706287545308	0.2882855490015857	T	T	T
0.2137728171667064	0.5007290549457224	0.4136168878109956	T	T	T
0.2957200099999966	0.0000000000000000	0.1054000099999968	F	F	F
0.3054200199999997	0.0000000000000000	0.2256900100000010	F	F	F
0.2927532639385220	0.0015328570561834	0.3508650172517136	T	T	T
0.2999872892940516	0.0090589013168980	0.4808550853154188	T	T	T
0.8598053460369073	0.4999808220000190	0.9949306060429564	T	T	T
0.7982599899999983	0.5000000000000000	0.1207100000000025	F	F	F
0.8054199700000026	0.5000000000000000	0.2397100100000031	F	F	F
0.7904414219344176	0.5025807954442456	0.3678845921804310	T	T	T
0.7960928346601648	0.4058587488487788	0.4828899611994686	T	T	T
0.8277949756085121	0.1735990875776636	0.4603100085256742	T	T	T

※(100)- H^+-H^-

1.00000000000000

4.6612049999999998	0.0000000000000000	0.0000000000000000
0.0000000000000000	2.9619610000000001	0.0000000000000000
0.0000000000000000	0.0000000000000000	37.4114300000000028

Ti O H

8 16 2

Selective dynamics

Direct

0.9907499999999985	0.0000000000000000	0.0225500000000025	F	F	F
0.9933699899999979	0.0000000000000000	0.1430799899999968	F	F	F
0.0028612912214862	-0.0003139182572888	0.2646537534876320	T	T	T
0.0075179961818691	0.0016217905795053	0.3922282357808965	T	T	T
0.4921799899999968	0.5000000000000000	0.0828399999999974	F	F	F
0.4956800100000009	0.5000000000000000	0.2028299999999987	F	F	F
0.5013659490549415	0.5009381176514922	0.3287214737514866	T	T	T
0.4572240503787776	0.5026287258795973	0.4635927348634230	T	T	T

0.6989700000000028 0.0000000000000000 0.0618600000000029 F F F
0.6879699699999975 0.0000000000000000 0.1802099900000016 F F F
0.7051776296254213 0.0009813330521925 0.3047521000339962 T T T
0.7460826771206351 0.0023133635429394 0.4409974401710334 T T T
0.2021300099999976 0.5000000000000000 0.0441399999999987 F F F
0.1872499999999988 0.5000000000000000 0.1657300099999972 F F F
0.2051097555116824 0.5003782406052510 0.2887056790170166 T T T
0.2158324872999103 0.5020301233631420 0.4141551074964343 T T T
0.2957200099999966 0.0000000000000000 0.1054000099999968 F F F
0.3054200199999997 0.0000000000000000 0.2256900100000010 F F F
0.2880123085966793 0.0013762638593044 0.3515434686439303 T T T
0.2431394598492825 0.0024409933610196 0.4798526231953872 T T T
0.8586838442427086 0.4999970547835936 0.9950164769692075 T T T
0.7982599899999983 0.5000000000000000 0.1207100000000025 F F F
0.8054199700000026 0.5000000000000000 0.2397100100000031 F F F
0.7815875060260047 0.5017104061256668 0.3702404206528291 T T T
0.7060286485094713 0.5028286457710119 0.4969524636444890 T T T
0.9077771586233044 0.0024058628624221 0.4591490725146786 T T T

※(100)-TS2

1.00000000000000
4.6612000278999997 0.0000000000000000 0.0000000000000000
0.0000000000000000 2.9619999359199998 0.0000000000000000
0.0000000000000000 0.0000000000000000 37.4113999047799979
Ti O H
8 16 2
Selective dynamics
Direct
0.9907500011999986 0.0000000000000000 0.0225499993999989 F F F
0.9933699882000013 0.0000000000000000 0.1430799911999969 F F F
1.0065753520514304 -0.0039540959581146 0.2662879722223821 T T T
1.0117362574670539 0.9881273796521503 0.3939707473291828 T T T
0.4921799906000004 0.5000000000000000 0.0828400011999975 F F F
0.4956800076000007 0.5000000000000000 0.2028300011999988 F F F
0.5059013986845746 0.4917725149001270 0.3303600207251394 T T T
0.4714563826126399 0.4756629571246567 0.4616632698823036 T T T
0.6989700000000028 0.0000000000000000 0.0618599981999992 F F F
0.6879699694000010 0.0000000000000000 0.1802099912000017 F F F
0.7155179046476188 -0.0074488152290255 0.3070149762210121 T T T
0.7794178644219498 0.9703288390535164 0.4438074019764706 T T T
0.2021300106000012 0.5000000000000000 0.0441399999999987 F F F
0.1872499999999988 0.5000000000000000 0.1657300070000005 F F F
0.2162462710057747 0.4952221026899619 0.2895423194966213 T T T
0.2276903027985095 0.4879542736667680 0.4148340087892447 T T T

0.2957200069999999 0.0000000000000000 0.1054000070000001 F F F
0.3054200187999996 0.0000000000000000 0.2256900129999977 F F F
0.2895011411011921 0.9909245094493176 0.3525730008305497 T T T
0.2416459462686801 0.0060527056234896 0.4815691975516501 T T T
0.8754863946555957 0.5000181453155008 0.9947862472162683 F F F
0.7982599911999984 0.5000000000000000 0.1207100017999991 F F F
0.8033227002211365 0.4967750363497078 0.2425001014965980 T T T
0.7830783869731850 0.4884531178845889 0.3725803937872272 T T T
0.8681144244501643 0.4074716950344824 0.5044365991464141 T T T
0.9610823736187631 0.9826207396238967 0.4609091027796508 T T T

※(100)-H^+-H^+

1.00000000000000
4.6612000278999997 0.0000000000000000 0.0000000000000000
0.0000000000000000 2.9619999359199998 0.0000000000000000
0.0000000000000000 0.0000000000000000 37.4113999047799979
Ti O H
8 16 2
Selective dynamics
Direct
0.9907499999999985 0.0000000000000000 0.0225500000000025 F F F
0.9933699899999979 0.0000000000000000 0.1430799899999968 F F F
0.0026473327852120 -0.0006428428863722 0.2648929265493196 T T T
-0.0022779047899394 0.0178110152487355 0.3910305092372965 T T T
0.4921799899999968 0.5000000000000000 0.0828399999999974 F F F
0.4956800100000009 0.5000000000000000 0.2028299999999987 F F F
0.5011300894905220 0.4974382234831806 0.3291300498520111 T T T
0.4415607510879175 0.5174481499705975 0.4620546563707937 T T T
0.6989700000000028 0.0000000000000000 0.0618600000000029 F F F
0.6879699699999975 0.0000000000000000 0.1802099900000016 F F F
0.7086628511967378 -0.0028969033187366 0.3053849874004011 T T T
0.7415713187662428 0.0179491445823724 0.4524695667777170 T T T
0.2021300099999976 0.5000000000000000 0.0441399999999987 F F F
0.1872499999999988 0.5000000000000000 0.1657300099999972 F F F
0.2060088778815594 0.4986398066246913 0.2886131140239399 T T T
0.1990788008317071 0.5265578074961974 0.4225180759289424 T T T
0.2957200099999966 0.0000000000000000 0.1054000099999968 F F F
0.3054200199999997 0.0000000000000000 0.2256900100000010 F F F
0.2851090474285306 -0.0045813832243775 0.3515583058957079 T T T
0.2890403607519770 0.0159104678864128 0.4968432095202697 T T T
0.8607111823592581 0.4999970462218259 0.9946754526871907 T T T
0.7982599899999983 0.5000000000000000 0.1207100000000025 F F F
0.8054199700000026 0.5000000000000000 0.2397100100000031 F F F
0.7500466249400053 0.5040745747767843 0.3726971790233209 T T T

```
0.1526931598909132   0.0144835270470761   0.5163804034822491   T   T   T
0.9382620017255254   0.0168969252990854   0.4614896726355668   T   T   T
```

※(110)-bare

```
1.00000000000000
  6.0177998542999998   0.0000000000000000   0.0000000000000000
  0.0000000000000000  13.0965995788999994   0.0000000000000000
  0.0000000000000000   0.0000000000000000  24.1138000488000017
Ti   O   H
24   48   2
Selective dynamics
Direct
 0.0000000000000000   0.0000000000000000   0.0533199999999994   F   F   F
 0.0000000000000000   0.2500000000000000   0.1896899999999988   F   F   F
-0.0000775868934326  -0.0000449116615511   0.3224592158902428   T   T   T
 0.2500000000000000   0.2500000000000000   0.0533199999999994   F   F   F
 0.2500000000000000   0.0000000000000000   0.1896899999999988   F   F   F
 0.2500242127595584   0.2499296515469911   0.3320854445773209   T   T   T
 0.5000000000000000   0.0000000000000000   0.0533199999999994   F   F   F
 0.5000000000000000   0.2500000000000000   0.1896899999999988   F   F   F
 0.4999211604893551  -0.0000100350950193   0.3224813362258777   T   T   T
 0.7499999799999983   0.2500000000000000   0.0533199999999994   F   F   F
 0.7499999799999983   0.0000000000000000   0.1896899999999988   F   F   F
 0.7497436096257363   0.2498910599700177   0.3320373240029290   T   T   T
 0.0000000000000000   0.5000000000000000   0.0533199999999994   F   F   F
 0.0000000000000000   0.7500000200000017   0.1896899999999988   F   F   F
-0.0001328749367197   0.4998189772262316   0.3234387110501082   T   T   T
 0.2500000000000000   0.7500000200000017   0.0533199999999994   F   F   F
 0.2500000000000000   0.5000000000000000   0.1896899999999988   F   F   F
 0.2499780792751334   0.7500160253872313   0.3320507282559009   T   T   T
 0.5000000000000000   0.5000000000000000   0.0533199999999994   F   F   F
 0.5000000000000000   0.7500000200000017   0.1896899999999988   F   F   F
 0.4998679542952505   0.5000605795961403   0.3219445985191813   T   T   T
 0.7499999799999983   0.7500000200000017   0.0533199999999994   F   F   F
 0.7499999799999983   0.5000000000000000   0.1896899999999988   F   F   F
 0.7497627628562566   0.7499824373313994   0.3320961156682768   T   T   T
 0.0000000000000000   0.2500000000000000   0.0000000000000000   F   F   F
 0.0000000000000000   0.0000000000000000   0.1363700100000003   F   F   F
-0.0000162360318745   0.2489313673895272   0.2722441947647348   T   T   T
 0.0000000000000000   0.2500000000000000   0.1066399999999987   F   F   F
 0.0000000000000000   0.0000000000000000   0.2430100099999990   F   F   F
-0.0001677786159065   0.2515317151768287   0.3794957991374647   T   T   T
 0.2500000000000000   0.0977499999999978   0.0533199999999994   F   F   F
 0.2500000000000000   0.3477499899999970   0.1896899999999988   F   F   F
```

0.2498296843677858	0.0928635635564961	0.3345720014163551	T	T	T
0.2500000000000000	0.4022499900000014	0.0533199999999994	F	F	F
0.2500000000000000	0.1522500100000030	0.1896899999999988	F	F	F
0.2509582757255849	0.4066425968300953	0.3330954285475348	T	T	T
0.5000000000000000	0.2500000000000000	0.0000000000000000	F	F	F
0.5000000000000000	0.0000000000000000	0.1363700100000003	F	F	F
0.4998805006601356	0.2490429658581801	0.2722458571491644	T	T	T
0.5000000000000000	0.2500000000000000	0.1066399999999987	F	F	F
0.5000000000000000	0.0000000000000000	0.2430100099999990	F	F	F
0.4999544299450802	0.2505324306464229	0.3794995748743392	T	T	T
0.7499999799999983	0.0977499999999978	0.0533199999999994	F	F	F
0.7499999799999983	0.3477499899999970	0.1896899999999988	F	F	F
0.7499991492872813	0.0928679610103199	0.3345115521690589	T	T	T
0.7499999799999983	0.4022499900000014	0.0533199999999994	F	F	F
0.7499999799999983	0.1522500100000030	0.1896899999999988	F	F	F
0.7488357898696016	0.4066147145335462	0.3330509239332731	T	T	T
0.0000000000000000	0.7500000200000017	0.0000000000000000	F	F	F
0.0000000000000000	0.5000000000000000	0.1363700100000003	F	F	F
-0.0001349711397927	0.7509903184172017	0.2722503796817322	T	T	T
0.0000000000000000	0.7500000200000017	0.1066399999999987	F	F	F
0.0000000000000000	0.5000000000000000	0.2430100099999990	F	F	F
-0.0000727791357524	0.7485802246195683	0.3795065520610351	T	T	T
0.2500000000000000	0.5977500099999986	0.0533199999999994	F	F	F
0.2500000000000000	0.8477499899999970	0.1896899999999988	F	F	F
0.2505897701763727	0.5932497279834770	0.3331916698765112	T	T	T
0.2500000000000000	0.9022500299999976	0.0533199999999994	F	F	F
0.2500000000000000	0.6522499699999997	0.1896899999999988	F	F	F
0.2498895558456996	0.9070717771939633	0.3344683798625074	T	T	T
0.5000000000000000	0.7500000200000017	0.0000000000000000	F	F	F
0.5000000000000000	0.5000000000000000	0.1363700100000003	F	F	F
0.4999417224554515	0.7507899612361756	0.2722539450926812	T	T	T
0.5000000000000000	0.7500000200000017	0.1066399999999987	F	F	F
0.5000000000000000	0.5000000000000000	0.2430100099999990	F	F	F
0.4998195438077417	0.7494291119886612	0.3795003596775239	T	T	T
0.7499999799999983	0.5977500099999986	0.0533199999999994	F	F	F
0.7499999799999983	0.8477499899999970	0.1896899999999988	F	F	F
0.7490837630986528	0.5932286853378935	0.3332070211372793	T	T	T
0.7499999799999983	0.9022500299999976	0.0533199999999994	F	F	F
0.7499999799999983	0.6522499699999997	0.1896899999999988	F	F	F
0.7499312977603746	0.9070771776207694	0.3345327753749677	T	T	T
-0.0042302189024367	0.4700569025728925	0.4247191666850267	T	T	T
0.0026501448102331	0.5281293111552626	0.4243659912613063	T	T	T

※(110)-H_2

```
 1.00000000000000
   5.9239001273999996    0.0000000000000000    0.0000000000000000
   0.0000000000000000   13.1838998795000002    0.0000000000000000
   0.0000000000000000    0.0000000000000000   27.4654006957999997
 Ti   O    H
  32   64    2
Selective dynamics
Direct
 0.0000000000000000  0.2500000000000000  0.0469200009999966   F   F   F
 0.0000000000000000  0.0000000000000000  0.1669299980000005   F   F   F
 0.9997650858038083  0.2490600211728926  0.2927006030425817   T   T   T
 0.9992658344881975  0.9981279234079629  0.4059138792555976   T   T   T
 0.2500000000000000  0.0000000000000000  0.0469200009999966   F   F   F
 0.2500000000000000  0.2500000000000000  0.1669299980000005   F   F   F
 0.2499334687660536  0.9993231366774450  0.2871316267217316   T   T   T
 0.2496607065985710  0.2480240089055323  0.4167822305416769   T   T   T
 0.5000000000000000  0.2500000000000000  0.0469200009999966   F   F   F
 0.5000000000000000  0.0000000000000000  0.1669299980000005   F   F   F
 0.4998662191178251  0.2490542756406270  0.2926426775613734   T   T   T
 0.4992973668871166  0.9982693640878324  0.4059670051882403   T   T   T
 0.7499999799999983  0.0000000000000000  0.0469200009999966   F   F   F
 0.7499999799999983  0.2500000000000000  0.1669299980000005   F   F   F
 0.7499282320393709  0.9992206645134259  0.2871374574831126   T   T   T
 0.7493639256728251  0.2480293747344503  0.4167819844882731   T   T   T
 0.0000000000000000  0.7500000180000015  0.0469200009999966   F   F   F
 0.0000000000000000  0.5000000000000000  0.1669299980000005   F   F   F
 0.9996719373999641  0.7492240825315264  0.2928066441877181   T   T   T
 0.9994580539621433  0.4977240258756412  0.4069636877245465   T   T   T
 0.2500000000000000  0.5000000000000000  0.0469200009999966   F   F   F
 0.2500000000000000  0.7500000180000015  0.1669299980000005   F   F   F
 0.2494124282086289  0.4992386752848635  0.2873315036755278   T   T   T
 0.2495869481742814  0.7482791385336788  0.4169879134529866   T   T   T
 0.5000000000000000  0.7500000180000015  0.0469200009999966   F   F   F
 0.5000000000000000  0.5000000000000000  0.1669299980000005   F   F   F
 0.4995162060676895  0.7491070393005802  0.2927446239993722   T   T   T
 0.4994089060862924  0.4985217599301655  0.4053834679768203   T   T   T
 0.7499999799999983  0.5000000000000000  0.0469200009999966   F   F   F
 0.7499999799999983  0.7500000180000015  0.1669299980000005   F   F   F
 0.7504666681165306  0.4993993231117726  0.2872781562580295   T   T   T
 0.7494868334290736  0.7482757226412560  0.4169848188796969   T   T   T
 0.0000000000000000  0.0000000000000000  0.0000000000000000   F   F   F
 0.0000000000000000  0.2500000000000000  0.1199999930000004   F   F   F
 0.9999962666725211  0.9991573209725171  0.2419738856406450   T   T   T
 0.9995150022765417  0.2469926757516491  0.3632156529767125   T   T   T
```

0.0000000000000000	0.0000000000000000	0.0938400009999967	F	F	F
0.0000000000000000	0.2500000000000000	0.2138500029999975	F	F	F
0.9999048746915419	0.9993772947307722	0.3372718965976630	T	T	T
-0.0005463160526877	0.2501423831261776	0.4587326134730140	T	T	T
0.2500000000000000	0.3477499910000006	0.0469200009999966	F	F	F
0.2500000000000000	0.0977499999999978	0.1669299980000005	F	F	F
0.2497504481245396	0.3483918295849674	0.2895921833093652	T	T	T
0.2490829628458540	0.0919735871918489	0.4179436473028913	T	T	T
0.2500000000000000	0.1522500089999994	0.0469200009999966	F	F	F
0.2500000000000000	0.4022499909999979	0.1669299980000005	F	F	F
0.2497913915732324	0.1498643462842565	0.2888768902446301	T	T	T
0.2511832223328789	0.4036356195618541	0.4158384979932339	T	T	T
0.5000000000000000	0.0000000000000000	0.0000000000000000	F	F	F
0.5000000000000000	0.2500000000000000	0.1199999930000004	F	F	F
0.4999831565566761	0.9991767673997415	0.2419768490303311	T	T	T
0.4995083462627366	0.2469115952708215	0.3631840544269974	T	T	T
0.5000000000000000	0.0000000000000000	0.0938400009999967	F	F	F
0.5000000000000000	0.2500000000000000	0.2138500029999975	F	F	F
0.4998933904405592	0.9994720366429661	0.3373133977395505	T	T	T
0.4995358806162480	0.2489727336543812	0.4586904490992542	T	T	T
0.7499999799999983	0.3477499910000006	0.0469200009999966	F	F	F
0.7499999799999983	0.0977499999999978	0.1669299980000005	F	F	F
0.7498007817841316	0.3484499037354407	0.2896174084198049	T	T	T
0.7495732567814199	0.0919584981490583	0.4179217449541984	T	T	T
0.7499999799999983	0.1522500089999994	0.0469200009999966	F	F	F
0.7499999799999983	0.4022499909999979	0.1669299980000005	F	F	F
0.7498194296971029	0.1498474714003933	0.2888836216453887	T	T	T
0.7476951641718634	0.4036094434595020	0.4157974268043468	T	T	T
0.0000000000000000	0.5000000000000000	0.0000000000000000	F	F	F
0.0000000000000000	0.7500000180000015	0.1199999930000004	F	F	F
-0.0000448389187090	0.4992126818894966	0.2420589608039601	T	T	T
0.9996064287166728	0.7486741496251210	0.3633197651052458	T	T	T
0.0000000000000000	0.5000000000000000	0.0938400009999967	F	F	F
0.0000000000000000	0.7500000180000015	0.2138500029999975	F	F	F
-0.0000890334075733	0.4996594657759940	0.3376431479224771	T	T	T
0.9996064706453784	0.7482270360083831	0.4588554360635816	T	T	T
0.2500000000000000	0.8477499910000006	0.0469200009999966	F	F	F
0.2500000000000000	0.5977500090000021	0.1669299980000005	F	F	F
0.2497584005125647	0.8486815260342111	0.2894953752524000	T	T	T
0.2498896856640215	0.5921205521821447	0.4174478640818243	T	T	T
0.2500000000000000	0.6522499729999964	0.0469200009999966	F	F	F
0.2500000000000000	0.9022500270000009	0.1669299980000005	F	F	F
0.2495210381179040	0.6500953349099871	0.2890469488499843	T	T	T
0.2495017174046688	0.9042054850570821	0.4172294036888083	T	T	T

0.5000000000000000	0.5000000000000000	0.0000000000000000	F	F	F
0.5000000000000000	0.7500000180000015	0.1199999930000004	F	F	F
0.4998418646592696	0.4992190668372994	0.2420548263457651	T	T	T
0.4995186264254389	0.7483439584264190	0.3632851256806771	T	T	T
0.5000000000000000	0.5000000000000000	0.0938400009999967	F	F	F
0.5000000000000000	0.7500000180000015	0.2138500029999975	F	F	F
0.4999779760805734	0.4999574701246471	0.3369274740327676	T	T	T
0.4996132304908417	0.7485978839721052	0.4588308033821569	T	T	T
0.7499999799999983	0.8477499910000006	0.0469200009999966	F	F	F
0.7499999799999983	0.5977500090000021	0.1669299980000005	F	F	F
0.7494996080983114	0.8486257433421768	0.2894934210937400	T	T	T
0.7490246148849302	0.5921213526122594	0.4174244621491003	T	T	T
0.7499999799999983	0.6522499729999964	0.0469200009999966	F	F	F
0.7499999799999983	0.9022500270000009	0.1669299980000005	F	F	F
0.7498591085225698	0.6501454313357538	0.2890434313198271	T	T	T
0.7492470899703237	0.9042226211613336	0.4172431251987817	T	T	T
0.9978870385086049	0.5066522534583000	0.5012362159394096	T	T	T
0.9982894426571359	0.4494964413405536	0.4978527311914962	T	T	T

※(110)-TS1

1.00000000000000

6.0177998542999998	0.0000000000000000	0.0000000000000000
0.0000000000000000	13.0965995788999994	0.0000000000000000
0.0000000000000000	0.0000000000000000	24.1138000488000017

Ti O H

24 48 2

Selective dynamics

Direct

0.0000000000000000	0.0000000000000000	0.0533199999999994	F	F	F
0.0000000000000000	0.2500000000000000	0.1896899999999988	F	F	F
0.0002089637339929	-0.0000977958285672	0.3225969244433243	T	T	T
0.2500000000000000	0.2500000000000000	0.0533199999999994	F	F	F
0.2500000000000000	0.0000000000000000	0.1896899999999988	F	F	F
0.2497499552764376	0.2504748403313298	0.3324158002131151	T	T	T
0.5000000000000000	0.0000000000000000	0.0533199999999994	F	F	F
0.5000000000000000	0.2500000000000000	0.1896899999999988	F	F	F
0.5002131218235776	0.9964856211066111	0.3228576931278021	T	T	T
0.7499999799999983	0.2500000000000000	0.0533199999999994	F	F	F
0.7499999799999983	0.0000000000000000	0.1896899999999988	F	F	F
0.7508721785560344	0.2504816900276063	0.3324070205424114	T	T	T
0.0000000000000000	0.5000000000000000	0.0533199999999994	F	F	F
0.0000000000000000	0.7500000200000017	0.1896899999999988	F	F	F
0.0002960431490128	0.5050917388910623	0.3289442821809787	T	T	T
0.2500000000000000	0.7500000200000017	0.0533199999999994	F	F	F

0.2500000000000000	0.5000000000000000	0.1896899999999988	F	F	F
0.2562288601055870	0.7469808158820171	0.3308722075453917	T	T	T
0.5000000000000000	0.5000000000000000	0.0533199999999994	F	F	F
0.5000000000000000	0.7500000200000017	0.1896899999999988	F	F	F
0.5002780979295649	0.4939037004308185	0.3213559720595240	T	T	T
0.7499999799999983	0.7500000200000017	0.0533199999999994	F	F	F
0.7499999799999983	0.5000000000000000	0.1896899999999988	F	F	F
0.7443289431532552	0.7469803137551428	0.3308713275245191	T	T	T
0.0000000000000000	0.2500000000000000	0.0000000000000000	F	F	F
0.0000000000000000	0.0000000000000000	0.1363700100000003	F	F	F
0.0001954020651905	0.2480181465142305	0.2725164325447149	T	T	T
0.0000000000000000	0.2500000000000000	0.1066399999999987	F	F	F
0.0000000000000000	0.0000000000000000	0.2430100099999990	F	F	F
0.0002706154947082	0.2454870652229588	0.3797359210344537	T	T	T
0.2500000000000000	0.0977499999999978	0.0533199999999994	F	F	F
0.2500000000000000	0.3477499899999970	0.1896899999999988	F	F	F
0.2536413181583054	0.0904722294046555	0.3338087251873418	T	T	T
0.2500000000000000	0.4022499900000014	0.0533199999999994	F	F	F
0.2500000000000000	0.1522500100000030	0.1896899999999988	F	F	F
0.2453401905555347	0.4068227623919187	0.3360740498061241	T	T	T
0.5000000000000000	0.2500000000000000	0.0000000000000000	F	F	F
0.5000000000000000	0.0000000000000000	0.1363700100000003	F	F	F
0.5001639119703116	0.2515798287499724	0.2725122822769552	T	T	T
0.5000000000000000	0.2500000000000000	0.1066399999999987	F	F	F
0.5000000000000000	0.0000000000000000	0.2430100099999990	F	F	F
0.5002913677788581	0.2487172654475941	0.3795443958677045	T	T	T
0.7499999799999983	0.0977499999999978	0.0533199999999994	F	F	F
0.7499999799999983	0.3477499899999970	0.1896899999999988	F	F	F
0.7468215085587422	0.0904882536850501	0.3337594403846654	T	T	T
0.7499999799999983	0.4022499900000014	0.0533199999999994	F	F	F
0.7499999799999983	0.1522500100000030	0.1896899999999988	F	F	F
0.7552342366320510	0.4068212183254600	0.3360667500512839	T	T	T
0.0000000000000000	0.7500000200000017	0.0000000000000000	F	F	F
0.0000000000000000	0.5000000000000000	0.1363700100000003	F	F	F
0.0001610918095229	0.7547922126991045	0.2742774418484362	T	T	T
0.0000000000000000	0.7500000200000017	0.1066399999999987	F	F	F
0.0000000000000000	0.5000000000000000	0.2430100099999990	F	F	F
0.0002921814837595	0.7202773184683646	0.3829758556771187	T	T	T
0.2500000000000000	0.5977500099999986	0.0533199999999994	F	F	F
0.2500000000000000	0.8477499899999970	0.1896899999999988	F	F	F
0.2652317859665242	0.5951857855773882	0.3260684573073142	T	T	T
0.2500000000000000	0.9022500299999976	0.0533199999999994	F	F	F
0.2500000000000000	0.6522499699999997	0.1896899999999988	F	F	F
0.2453715751558663	0.9036678462050919	0.3370325862030191	T	T	T

0.5000000000000000	0.7500000200000017	0.0000000000000000	F	F	F
0.5000000000000000	0.5000000000000000	0.1363700100000003	F	F	F
0.5002236885318732	0.7551735084497868	0.2734083878233091	T	T	T
0.5000000000000000	0.7500000200000017	0.1066399999999987	F	F	F
0.5000000000000000	0.5000000000000000	0.2430100099999990	F	F	F
0.5002550371319117	0.7478695412809507	0.3799151674323549	T	T	T
0.7499999799999983	0.5977500099999986	0.0533199999999994	F	F	F
0.7499999799999983	0.8477499899999970	0.1896899999999988	F	F	F
0.7353177368763789	0.5951770657988338	0.3261101304375507	T	T	T
0.7499999799999983	0.9022500299999976	0.0533199999999994	F	F	F
0.7499999799999983	0.6522499699999997	0.1896899999999988	F	F	F
0.7551026778127897	0.9036832627014063	0.3370768611424558	T	T	T
0.0006623935799739	0.5514926494804819	0.4023646153079649	T	T	T
0.0003488726262579	0.6376438524312051	0.3965269767189566	T	T	T

※(110)-H^{+}-H^{-}

1.00000000000000

5.9239001273999996	0.0000000000000000	0.0000000000000000
0.0000000000000000	13.1838998795000002	0.0000000000000000
0.0000000000000000	0.0000000000000000	27.4654006957999997

Ti O H

32 64 2

Selective dynamics

Direct

0.0000000000000000	0.2500000000000000	0.0469200009999966	F	F	F
0.0000000000000000	0.0000000000000000	0.1669299980000005	F	F	F
0.9995565857383447	0.2493468578939986	0.2918126320895935	T	T	T
0.9993328654396552	0.0004812233239241	0.4057343306699323	T	T	T
0.2500000000000000	0.0000000000000000	0.0469200009999966	F	F	F
0.2500000000000000	0.2500000000000000	0.1669299980000005	F	F	F
0.2498692386614911	0.9990581073085216	0.2870275492720356	T	T	T
0.2582671929860709	0.2509161327629215	0.4131518448789995	T	T	T
0.5000000000000000	0.2500000000000000	0.0469200009999966	F	F	F
0.5000000000000000	0.0000000000000000	0.1669299980000005	F	F	F
0.4995753486783842	0.2497776319650068	0.2885377638009195	T	T	T
0.4993637742615585	0.0014618540835418	0.4060623880585330	T	T	T
0.7499999799999983	0.0000000000000000	0.0469200009999966	F	F	F
0.7499999799999983	0.2500000000000000	0.1669299980000005	F	F	F
0.7498630433694259	0.9990574260464297	0.2870216872410089	T	T	T
0.7407178456129812	0.2509812449119457	0.4131955864700477	T	T	T
0.0000000000000000	0.7500000180000015	0.0469200009999966	F	F	F
0.0000000000000000	0.5000000000000000	0.1669299980000005	F	F	F
0.9992555222175911	0.7500641977506025	0.2928696696904650	T	T	T

0.9995000311580589	0.4998878355072344	0.4165098201341251	T	T	T
0.2500000000000000	0.5000000000000000	0.0469200009999966	F	F	F
0.2500000000000000	0.7500000180000015	0.1669299980000005	F	F	F
0.2442321004110299	0.5006302712040016	0.2878028406242842	T	T	T
0.2499820008418389	0.7495816417430687	0.4167549123719901	T	T	T
0.5000000000000000	0.7500000180000015	0.0469200009999966	F	F	F
0.5000000000000000	0.5000000000000000	0.1669299980000005	F	F	F
0.4992743752044106	0.7495602683807324	0.2924570720138106	T	T	T
0.4994173137002954	0.5006132235642539	0.4040201863499905	T	T	T
0.7499999799999983	0.5000000000000000	0.0469200009999966	F	F	F
0.7499999799999983	0.7500000180000015	0.1669299980000005	F	F	F
0.7548721347103915	0.5005839050907225	0.2878718359582669	T	T	T
0.7488295570817286	0.7495260597599886	0.4168192779440017	T	T	T
0.0000000000000000	0.0000000000000000	0.0000000000000000	F	F	F
0.0000000000000000	0.2500000000000000	0.1199999930000004	F	F	F
0.9998671907452136	0.0003763918261373	0.2418687553233969	T	T	T
0.9994825419740055	0.2455725384027994	0.3642070175147331	T	T	T
0.0000000000000000	0.0000000000000000	0.0938400009999967	F	F	F
0.0000000000000000	0.2500000000000000	0.2138500029999975	F	F	F
0.9996951035556001	0.0010387420740635	0.3371985349155315	T	T	T
0.9995661289876421	0.2546552349172050	0.4620643850994453	T	T	T
0.2500000000000000	0.3477499910000006	0.0469200009999966	F	F	F
0.2500000000000000	0.0977499999999978	0.1669299980000005	F	F	F
0.2501186720803887	0.3467430306893728	0.2894398352389493	T	T	T
0.2471513884357007	0.0963686485120344	0.4181429155451463	T	T	T
0.2500000000000000	0.1522500089999994	0.0469200009999966	F	F	F
0.2500000000000000	0.4022499909999979	0.1669299980000005	F	F	F
0.2497450139817370	0.1504368709711711	0.2887661267518151	T	T	T
0.2549646466648331	0.4022178640637506	0.4139793382898996	T	T	T
0.5000000000000000	0.0000000000000000	0.0000000000000000	F	F	F
0.5000000000000000	0.2500000000000000	0.1199999930000004	F	F	F
0.4998552382904468	0.0005094730778820	0.2418874576212909	T	T	T
0.4995133191607571	0.2462894495281459	0.3628781114318650	T	T	T
0.5000000000000000	0.0000000000000000	0.0938400009999967	F	F	F
0.5000000000000000	0.2500000000000000	0.2138500029999975	F	F	F
0.4997538752110946	0.0016326996775446	0.3374698354733018	T	T	T
0.4994609203776937	0.2503438827017231	0.4572455867082867	T	T	T
0.7499999799999983	0.3477499910000006	0.0469200009999966	F	F	F
0.7499999799999983	0.0977499999999978	0.1669299980000005	F	F	F
0.7490177006978649	0.3467371186911862	0.2894783505796648	T	T	T
0.7517688730922100	0.0963882392617540	0.4181655821999574	T	T	T
0.7499999799999983	0.1522500089999994	0.0469200009999966	F	F	F
0.7499999799999983	0.4022499909999979	0.1669299980000005	F	F	F
0.7494875479971574	0.1504444600677109	0.2887521417270710	T	T	T

0.7438068089171176	0.4022375164755360	0.4139847844386021	T	T	T
0.0000000000000000	0.5000000000000000	0.0000000000000000	F	F	F
0.0000000000000000	0.7500000180000015	0.1199999930000004	F	F	F
0.9995213403287211	0.4991331313022979	0.2409210428253425	T	T	T
0.9995487192621908	0.7518194475634309	0.3631764915131205	T	T	T
0.0000000000000000	0.5000000000000000	0.0938400009999967	F	F	F
0.0000000000000000	0.7500000180000015	0.2138500029999975	F	F	F
0.9996306026375934	0.4992937722719606	0.3354604981038723	T	T	T
0.9995343553415337	0.7512149388659458	0.4583845984316541	T	T	T
0.2500000000000000	0.8477499910000006	0.0469200009999966	F	F	F
0.2500000000000000	0.5977500090000021	0.1669299980000005	F	F	F
0.2497602520275417	0.8502395172353756	0.2890530442629781	T	T	T
0.2548103296139705	0.5945252980689278	0.4171394481894884	T	T	T
0.2500000000000000	0.6522499729999964	0.0469200009999966	F	F	F
0.2500000000000000	0.9022500270000009	0.1669299980000005	F	F	F
0.2493451862227850	0.6522067597756912	0.2888514555459056	T	T	T
0.2503883418778848	0.9079549323376861	0.4172326432666439	T	T	T
0.5000000000000000	0.5000000000000000	0.0000000000000000	F	F	F
0.5000000000000000	0.7500000180000015	0.1199999930000004	F	F	F
0.4997361310698453	0.4988652133845698	0.2417172676019049	T	T	T
0.4994634476424157	0.7511842973495553	0.3630414304693202	T	T	T
0.5000000000000000	0.5000000000000000	0.0938400009999967	F	F	F
0.5000000000000000	0.7500000180000015	0.2138500029999975	F	F	F
0.4994735907623767	0.5009818350812076	0.3360729279489701	T	T	T
0.4993816112934268	0.7528888846419253	0.4586304612033421	T	T	T
0.7499999799999983	0.8477499910000006	0.0469200009999966	F	F	F
0.7499999799999983	0.5977500090000021	0.1669299980000005	F	F	F
0.7492220796117871	0.8502401512964882	0.2890458104133445	T	T	T
0.7439440993981972	0.5945110305660567	0.4171238936343420	T	T	T
0.7499999799999983	0.6522499729999964	0.0469200009999966	F	F	F
0.7499999799999983	0.9022500270000009	0.1669299980000005	F	F	F
0.7495420304899537	0.6522002061257116	0.2888975759983724	T	T	T
0.7484891514690305	0.9079268510622199	0.4172354699344965	T	T	T
0.9992579809210048	0.4823501826443620	0.4784356804782550	T	T	T
0.9996131222680479	0.3145367613598974	0.4832433638949959	T	T	T

※(110)-TS2

1.00000000000000

6.0177998542999998	0.0000000000000000	0.0000000000000000
0.0000000000000000	13.0965995788999994	0.0000000000000000
0.0000000000000000	0.0000000000000000	24.1138000488000017

Ti O H

24 48 2

Selective dynamics

Direct

0.0000000000000000	0.0000000000000000	0.0533199999999994	F	F	F
0.0000000000000000	0.2500000000000000	0.1896899999999988	F	F	F
0.0002288837862940	1.0001168831409133	0.3224239425003571	T	T	T
0.2500000000000000	0.2500000000000000	0.0533199999999994	F	F	F
0.2500000000000000	0.0000000000000000	0.1896899999999988	F	F	F
0.2502409915849837	0.2511197457191681	0.3321755549030255	T	T	T
0.5000000000000000	0.0000000000000000	0.0533199999999994	F	F	F
0.5000000000000000	0.2500000000000000	0.1896899999999988	F	F	F
0.5002314128377023	-0.0014701325104042	0.3228050198743537	T	T	T
0.7499999799999983	0.2500000000000000	0.0533199999999994	F	F	F
0.7499999799999983	0.0000000000000000	0.1896899999999988	F	F	F
0.7504241557920955	0.2511259928027766	0.3321536502947823	T	T	T
0.0000000000000000	0.5000000000000000	0.0533199999999994	F	F	F
0.0000000000000000	0.7500000200000017	0.1896899999999988	F	F	F
0.0002459192146160	0.5020287062659298	0.3323402063087090	T	T	T
0.2500000000000000	0.7500000200000017	0.0533199999999994	F	F	F
0.2500000000000000	0.5000000000000000	0.1896899999999988	F	F	F
0.2541031792855828	0.7463495369370556	0.3327126941045070	T	T	T
0.5000000000000000	0.5000000000000000	0.0533199999999994	F	F	F
0.5000000000000000	0.7500000200000017	0.1896899999999988	F	F	F
0.5002487634402222	0.4974877071210034	0.3211612703614409	T	T	T
0.7499999799999983	0.7500000200000017	0.0533199999999994	F	F	F
0.7499999799999983	0.5000000000000000	0.1896899999999988	F	F	F
0.7467279330520291	0.7463420062047744	0.3327636313584037	T	T	T
0.0000000000000000	0.2500000000000000	0.0000000000000000	F	F	F
0.0000000000000000	0.0000000000000000	0.1363700100000003	F	F	F
0.0002279877525782	0.2478440247175018	0.2723056408963246	T	T	T
0.0000000000000000	0.2500000000000000	0.1066399999999987	F	F	F
0.0000000000000000	0.0000000000000000	0.2430100099999990	F	F	F
0.0002698121726911	0.2489940174267014	0.3794229122402276	T	T	T
0.2500000000000000	0.0977499999999978	0.0533199999999994	F	F	F
0.2500000000000000	0.3477499899999970	0.1896899999999988	F	F	F
0.2522068689423617	0.0913229305625688	0.3341981789268046	T	T	T
0.2500000000000000	0.4022499900000014	0.0533199999999994	F	F	F
0.2500000000000000	0.1522500100000030	0.1896899999999988	F	F	F
0.2499974546358778	0.4067381017972547	0.3344985466201389	T	T	T
0.5000000000000000	0.2500000000000000	0.0000000000000000	F	F	F
0.5000000000000000	0.0000000000000000	0.1363700100000003	F	F	F
0.5001551179017308	0.2493462584879908	0.2722985307930571	T	T	T
0.5000000000000000	0.2500000000000000	0.1066399999999987	F	F	F
0.5000000000000000	0.0000000000000000	0.2430100099999990	F	F	F
0.5003357188890823	0.2486327722151670	0.3793975240498482	T	T	T
0.7499999799999983	0.0977499999999978	0.0533199999999994	F	F	F

0.7499999799999983	0.3477499899999970	0.1896899999999988	F	F	F
0.7482945982649055	0.0913508783183441	0.3341133968103486	T	T	T
0.7499999799999983	0.4022499900000014	0.0533199999999994	F	F	F
0.7499999799999983	0.1522500100000030	0.1896899999999988	F	F	F
0.7505737078025769	0.4067319177861359	0.3344370330647163	T	T	T
0.0000000000000000	0.7500000200000017	0.0000000000000000	F	F	F
0.0000000000000000	0.5000000000000000	0.1363700100000003	F	F	F
0.0001880206977217	0.7540940225121394	0.2759122878974769	T	T	T
0.0000000000000000	0.7500000200000017	0.1066399999999987	F	F	F
0.0000000000000000	0.5000000000000000	0.2430100099999990	F	F	F
0.0004219771187809	0.7120113423882795	0.3865689712178133	T	T	T
0.2500000000000000	0.5977500099999986	0.0533199999999994	F	F	F
0.2500000000000000	0.8477499899999970	0.1896899999999988	F	F	F
0.2615806913825752	0.5946689475519912	0.3295422270702251	T	T	T
0.2500000000000000	0.9022500299999976	0.0533199999999994	F	F	F
0.2500000000000000	0.6522499699999997	0.1896899999999988	F	F	F
0.2460417329743605	0.9041692475298998	0.3360452886810831	T	T	T
0.5000000000000000	0.7500000200000017	0.0000000000000000	F	F	F
0.5000000000000000	0.5000000000000000	0.1363700100000003	F	F	F
0.5002689742006776	0.7515633557257689	0.2744713233956783	T	T	T
0.5000000000000000	0.7500000200000017	0.1066399999999987	F	F	F
0.5000000000000000	0.5000000000000000	0.2430100099999990	F	F	F
0.5003234499874591	0.7505395890516074	0.3808140142049066	T	T	T
0.7499999799999983	0.5977500099999986	0.0533199999999994	F	F	F
0.7499999799999983	0.8477499899999970	0.1896899999999988	F	F	F
0.7389384083663514	0.5946823938400954	0.3295554270182576	T	T	T
0.7499999799999983	0.9022500299999976	0.0533199999999994	F	F	F
0.7499999799999983	0.6522499699999997	0.1896899999999988	F	F	F
0.7544968371241941	0.9041985229564391	0.3361286314692927	T	T	T
1.0000227620727358	0.5391938407351967	0.4075556369225718	T	T	T
0.0003903057272182	0.6290694961522478	0.4016733791121966	T	T	T

※(110)-H⁺-H⁺

1.00000000000000

6.0177998542999998	0.0000000000000000	0.0000000000000000
0.0000000000000000	13.0965995788999994	0.0000000000000000
0.0000000000000000	0.0000000000000000	24.1138000488000017

Ti O H
24 48 2
Selective dynamics
Direct

0.0000000000000000	0.0000000000000000	0.0533200030000032	F	F	F
0.0000000000000000	0.2500000000000000	0.1896900010000024	F	F	F
-0.0000427489681594	0.0000640850535770	0.3209621480107626	T	T	T

0.2500000000000000	0.2500000000000000	0.0533200030000032	F	F	F
0.2500000000000000	0.0000000000000000	0.1896900010000024	F	F	F
0.2584098721422534	0.2505105061284917	0.3313310969801091	T	T	T
0.5000000000000000	0.0000000000000000	0.0533200030000032	F	F	F
0.5000000000000000	0.2500000000000000	0.1896900010000024	F	F	F
0.4998571052880277	-0.0000396882681792	0.3213039786235906	T	T	T
0.7500000200000017	0.2500000000000000	0.0533200030000032	F	F	F
0.7500000200000017	0.0000000000000000	0.1896900010000024	F	F	F
0.7390340656745266	0.2512658910852243	0.3314030660970853	T	T	T
0.0000000000000000	0.5000000000000000	0.0533200030000032	F	F	F
0.0000000000000000	0.7500000000000000	0.1896900010000024	F	F	F
-0.0002553110377566	0.4999742626215292	0.3263848539588211	T	T	T
0.2500000000000000	0.7500000000000000	0.0533200030000032	F	F	F
0.2500000000000000	0.5000000000000000	0.1896900010000024	F	F	F
0.2605731838846220	0.7486522863157082	0.3313298780870391	T	T	T
0.5000000000000000	0.5000000000000000	0.0533200030000032	F	F	F
0.5000000000000000	0.7500000000000000	0.1896900010000024	F	F	F
0.4999703023110693	0.5001623419575443	0.3225327787204949	T	T	T
0.7500000200000017	0.7500000000000000	0.0533200030000032	F	F	F
0.7500000200000017	0.5000000000000000	0.1896900010000024	F	F	F
0.7413456263442851	0.7495139483356582	0.3313665120289309	T	T	T
0.0000000000000000	0.2500000000000000	0.0000000000000000	F	F	F
0.0000000000000000	0.0000000000000000	0.1363700030000032	F	F	F
-0.0008048378871461	0.2503121864983393	0.2775228052241938	T	T	T
0.0000000000000000	0.2500000000000000	0.1066400059999992	F	F	F
0.0000000000000000	0.0000000000000000	0.2430100079999988	F	F	F
-0.0008547978228902	0.2463846688338250	0.3866771506994539	T	T	T
0.2500000000000000	0.0977499999999978	0.0533200030000032	F	F	F
0.2500000000000000	0.3477500190000029	0.1896900010000024	F	F	F
0.2491951322625126	0.0942431077330185	0.3342256169336110	T	T	T
0.2500000000000000	0.4022499809999971	0.0533200030000032	F	F	F
0.2500000000000000	0.1522500089999994	0.1896900010000024	F	F	F
0.2564159975397615	0.4044264974331243	0.3378836266218612	T	T	T
0.5000000000000000	0.2500000000000000	0.0000000000000000	F	F	F
0.5000000000000000	0.0000000000000000	0.1363700030000032	F	F	F
0.4980678460923964	0.2495293581477632	0.2751196014204284	T	T	T
0.5000000000000000	0.2500000000000000	0.1066400059999992	F	F	F
0.5000000000000000	0.0000000000000000	0.2430100079999988	F	F	F
0.4979654351584898	0.2454880243195163	0.3818253828757168	T	T	T
0.7500000200000017	0.0977499999999978	0.0533200030000032	F	F	F
0.7500000200000017	0.3477500190000029	0.1896900010000024	F	F	F
0.7500672981554641	0.0941688576203212	0.3342017247568521	T	T	T
0.7500000200000017	0.4022499809999971	0.0533200030000032	F	F	F
0.7500000200000017	0.1522500089999994	0.1896900010000024	F	F	F

0.7333917854009885	0.4024735086781243	0.3370793745525392	T	T	T
0.0000000000000000	0.7500000000000000	0.0000000000000000	F	F	F
0.0000000000000000	0.5000000000000000	0.1363700030000032	F	F	F
0.0007900947995292	0.7493341626993224	0.2775076548035470	T	T	T
0.0000000000000000	0.7500000000000000	0.1066400059999992	F	F	F
0.0000000000000000	0.5000000000000000	0.2430100079999988	F	F	F
0.0007348866916859	0.7542216192244234	0.3867026599411620	T	T	T
0.2500000000000000	0.5977500190000029	0.0533200030000032	F	F	F
0.2500000000000000	0.8477499820000034	0.1896900010000024	F	F	F
0.2659972025787686	0.5975069842366930	0.3375202426639918	T	T	T
0.2500000000000000	0.9022500179999966	0.0533200030000032	F	F	F
0.2500000000000000	0.6522500179999966	0.1896900010000024	F	F	F
0.2496127580136867	0.9057182543145648	0.3338758905009757	T	T	T
0.5000000000000000	0.7500000000000000	0.0000000000000000	F	F	F
0.5000000000000000	0.5000000000000000	0.1363700030000032	F	F	F
0.5017151411283456	0.7496969350761772	0.2751177257356573	T	T	T
0.5000000000000000	0.7500000000000000	0.1066400059999992	F	F	F
0.5000000000000000	0.5000000000000000	0.2430100079999988	F	F	F
0.5018226112661559	0.7551469327857456	0.3817606953565996	T	T	T
0.7500000200000017	0.5977500190000029	0.0533200030000032	F	F	F
0.7500000200000017	0.8477499820000034	0.1896900010000024	F	F	F
0.7437137480862499	0.5956097553567790	0.3384148205703031	T	T	T
0.7500000200000017	0.9022500179999966	0.0533200030000032	F	F	F
0.7500000200000017	0.6522500179999966	0.1896900010000024	F	F	F
0.7507239175983629	0.9056447372967853	0.3337539713799182	T	T	T
0.0012156652090079	0.2815334052956611	0.4221193755146013	T	T	T
-0.0004124549021207	0.7182380881719480	0.4218866853636188	T	T	T

※(101)-bare

1.00000000000000

5.5226850000000001	0.0000000000000000	0.0000000000000000
0.0000000000000000	4.6612049999999998	0.0000000000000000
0.0000000000000000	0.0000000000000000	29.3530349999999984

Ti O

8 16

Selective dynamics

Direct

0.0231342299999966	0.3381019999999992	0.0276814500000029	F	F	F
0.9956512099999983	0.9953572900000012	0.1147915199999971	F	F	F
0.0099299089697006	0.6776084152850740	0.2017730748388492	T	T	T
0.9526799730085348	0.3565620301880545	0.2857192583825442	T	T	T
0.4768657700000034	0.8381019999999992	0.0276814500000029	F	F	F
0.5043487900000017	0.4953572900000012	0.1147915199999971	F	F	F
0.4900700910302996	0.1776084152850741	0.2017730748388492	T	T	T

0.5473200269914652	0.8565620301880543	0.2857192583825442	T	T	T
0.6942687900000024	0.5390462800000009	0.0533909399999999	F	F	F
0.6888860199999982	0.2014475399999966	0.1424443100000019	F	F	F
0.6641690688406934	0.8326643606345397	0.2254899776376359	T	T	T
0.6684133517980543	0.5222471881598669	0.3115624886505791	T	T	T
0.1988094599999997	0.6164099100000016	0.0016000999999974	F	F	F
0.1904341199999990	0.2886050899999972	0.0893614800000009	F	F	F
0.1697858835153961	0.0064592048204837	0.1752014915343000	T	T	T
0.1494149219178352	0.6806262224977000	0.2626352756476690	T	T	T
0.3011905400000003	0.1164099100000016	0.0016000999999974	F	F	F
0.3095658800000010	0.7886050899999972	0.0893614800000009	F	F	F
0.3302141264846048	0.5064592048204838	0.1752014915343000	T	T	T
0.3505850780821648	0.1806262224977000	0.2626352756476690	T	T	T
0.8057312099999976	0.0390462800000009	0.0533909399999999	F	F	F
0.8111139800000018	0.7014475399999966	0.1424443100000019	F	F	F
0.8358309311593066	0.3326643606345394	0.2254899776376359	T	T	T
0.8315866482019457	0.0222471881598670	0.3115624886505791	T	T	T

***(101)-H_2**

1.00000000000000

5.5226998328999999	0.0000000000000000	0.0000000000000000
0.0000000000000000	4.6612000465000003	0.0000000000000000
0.0000000000000000	0.0000000000000000	29.3530006408999995

Ti O H
8 16 2
Selective dynamics
Direct

0.0231299989999982	0.3380999910000000	0.0276799999999966	F	F	F
0.9956499520000008	0.9953600139999992	0.1147900030000031	F	F	F
0.0054655434450658	0.6775277739893919	0.2013494812739868	T	T	T
0.9846508535543644	0.3409416763896612	0.2829106728778807	T	T	T
0.4768699819999966	0.8381000170000021	0.0276799999999966	F	F	F
0.5043500049999992	0.4953599629999985	0.1147900030000031	F	F	F
0.4886202470153407	0.1705648645615964	0.2021512172124643	T	T	T
0.5791716692513258	0.8652551829147739	0.2894077803506321	T	T	T
0.6942700279999983	0.5390499680000005	0.0533899989999966	F	F	F
0.6888899710000018	0.2014500020000014	0.1424400100000014	F	F	F
0.6726068487863864	0.8337774526421843	0.2270155342441172	T	T	T
0.6904090251257410	0.5171946336525594	0.3089993774829742	T	T	T
0.1988100049999986	0.6164100230000003	0.0016000000000034	F	F	F
0.1904299979999990	0.2886100039999988	0.0893600010000029	F	F	F
0.1741042216214712	0.0029206134665705	0.1748199042398473	T	T	T
0.1458045991873561	0.6815126933817330	0.2616119075710275	T	T	T

```
0.3011899950000014  0.1164099980000017  0.0016000000000034  F  F  F
0.3095700240000028  0.7886100039999988  0.0893600010000029  F  F  F
0.3344154806308295  0.5031966559800061  0.1757748457568185  T  T  T
0.3505594339180283  0.1748676134591436  0.2637105821581206  T  T  T
0.8057299720000017  0.0390500029999998  0.0533899989999966  F  F  F
0.8111099859999982  0.7014499889999968  0.1424400100000014  F  F  F
0.8461345602517906  0.3241631657801976  0.2244547879774966  T  T  T
0.8595290548290254  0.0246975355397191  0.3157381138380699  T  T  T
0.2837088635377858  0.9105357660974972  0.3509714544747282  T  T  T
0.4043356087368530  0.9208299031850584  0.3635080473168692  T  T  T
```

※(101)-H^+-H^-

```
   1.00000000000000
     5.5226998328999999    0.0000000000000000    0.0000000000000000
     0.0000000000000000    4.6612000465000003    0.0000000000000000
     0.0000000000000000    0.0000000000000000   29.3530006408999995
   Ti   O    H
     8    16    2
Selective dynamics
Direct
  0.0231299989999982  0.3380999910000000  0.0276799999999966  F  F  F
  0.9956499520000008  0.9953600139999992  0.1147900030000031  F  F  F
 -0.0133845153635041  0.6905696752129385  0.2027441244750440  T  T  T
  0.0217411438565838  0.3340919406241513  0.2826744369188228  T  T  T
  0.4768699819999966  0.8381000170000021  0.0276799999999966  F  F  F
  0.5043500049999992  0.4953599629999985  0.1147900030000031  F  F  F
  0.4914725924769892  0.1688944737378878  0.2006040608132500  T  T  T
  0.5500145316589903  0.8659907427762785  0.2919548814609807  T  T  T
  0.6942700279999983  0.5390499680000005  0.0533899989999966  F  F  F
  0.6888899710000018  0.2014500020000014  0.1424400100000014  F  F  F
  0.6564092807019870  0.8293147495993528  0.2256009833238274  T  T  T
  0.7401154831504491  0.5487819311211979  0.3196235867043034  T  T  T
  0.1988100049999986  0.6164100230000003  0.0016000000000034  F  F  F
  0.1904299979999990  0.2886100039999988  0.0893600010000029  F  F  F
  0.1748107991820639 -0.0003560656934251  0.1751510277818074  T  T  T
  0.1409177712769634  0.7030644161057198  0.2623478136236700  T  T  T
  0.3011899950000014  0.1164099980000017  0.0016000000000034  F  F  F
  0.3095700240000028  0.7886100039999988  0.0893600010000029  F  F  F
  0.3337613353056375  0.4994834113796479  0.1749639027041181  T  T  T
  0.3643398329946456  0.2065896195057450  0.2647121009803003  T  T  T
  0.8057299720000017  0.0390500029999998  0.0533899989999966  F  F  F
  0.8111099859999982  0.7014499889999968  0.1424400100000014  F  F  F
  0.8610577853812895  0.3217097386819194  0.2261834115380096  T  T  T
  0.8249251502178998  0.0554318987904823  0.3116392039829677  T  T  T
```

0.3620467467056658	0.9100567454701592	0.3394908168256388	T	T	T
0.6612563574744368	0.3822213251045740	0.3334004196422921	T	T	T

※(101)-TS1

1.00000000000000
5.5226998328999999 0.0000000000000000 0.0000000000000000
0.0000000000000000 4.6612000465000003 0.0000000000000000
0.0000000000000000 0.0000000000000000 29.3530006408999995
Ti O H
8 16 2
Selective dynamics
Direct

0.0231300000000019	0.3380999900000035	0.0276799999999966	F	F	F
0.9956499500000007	0.9953600099999989	0.1147899999999993	F	F	F
0.9879908249598156	0.6933098900291674	0.2023286975634172	T	T	T
0.9983945357848425	0.3536258874348959	0.2833029975715685	T	T	T
0.4768699800000036	0.8381000199999988	0.0276799999999966	F	F	F
0.5043500100000031	0.4953599600000018	0.1147899999999993	F	F	F
0.4825565114277861	0.1794008128658262	0.2014783880270676	T	T	T
0.5699757953493844	0.8968226165889474	0.2913550288801167	T	T	T
0.6942700299999984	0.5390499700000007	0.0533900000000003	F	F	F
0.6888899699999982	0.2014500000000012	0.1424400100000014	F	F	F
0.6581198205757859	0.8452381500748551	0.2271957680686524	T	T	T
0.6914552905265716	0.5352412873884327	0.3120650575284019	T	T	T
0.1988100100000025	0.6164100200000036	0.0016000000000034	F	F	F
0.1904299999999992	0.2886099999999985	0.0893599999999992	F	F	F
0.1698638289635281	0.0097808453016214	0.1751693578596006	T	T	T
0.1319424606088975	0.7063695124283963	0.2613986462413426	T	T	T
0.3011899899999975	0.1164100000000019	0.0016000000000034	F	F	F
0.3095700200000024	0.7886099999999985	0.0893599999999992	F	F	F
0.3334188524348053	0.5103326810906649	0.1752995717811499	T	T	T
0.3442329906288965	0.2002938308334896	0.2647856397129609	T	T	T
0.8057299700000016	0.0390500000000031	0.0533900000000003	F	F	F
0.8111099899999985	0.7014499900000004	0.1424400100000014	F	F	F
0.8447572888715337	0.3334532652407377	0.2260257419399775	T	T	T
0.8521317212916426	0.0487495948383092	0.3153698169477063	T	T	T
0.4047196945539994	0.7749368138522512	0.3466874474383832	T	T	T
0.5404406723227861	0.6156924976249000	0.3395443198980631	T	T	T

※(101)-TS2

1.00000000000000
5.5226998328999999 0.0000000000000000 0.0000000000000000
0.0000000000000000 4.6612000465000003 0.0000000000000000

```
      0.0000000000000000    0.0000000000000000   29.3530006408999995
   Ti   O   H
     8   16    2
Selective dynamics
Direct
  0.0231299989999982  0.3380999910000000  0.0276799999999966   F   F   F
  0.9956499520000008  0.9953600139999992  0.1147900030000031   F   F   F
  0.0012370609502403  0.6874002393934214  0.2021449487084943   T   T   T
  0.9833355381025782  0.3210374512065557  0.2841586953302587   T   T   T
  0.4768699819999966  0.8381000170000021  0.0276799999999966   F   F   F
  0.5043500049999992  0.4953599629999985  0.1147900030000031   F   F   F
  0.4988270433065193  0.1803134500093971  0.2013118577798925   T   T   T
  0.5053698836268093  0.8165037598118886  0.2840419592698382   T   T   T
  0.6942700279999983  0.5390499680000005  0.0533899989999966   F   F   F
  0.6888899710000018  0.2014500020000014  0.1424400100000014   F   F   F
  0.6711161251537613  0.8437674403904147  0.2241860943504116   T   T   T
  0.6869669745755336  0.5038128160944156  0.3181656158268557   T   T   T
  0.1988100049999986  0.6164100230000003  0.0016000000000034   F   F   F
  0.1904299979999990  0.2886100039999988  0.0893600010000029   F   F   F
  0.1688439073041851  0.0086534784956768  0.1755108327791723   T   T   T
  0.1647697018610439  0.6767516918017404  0.2628906090318323   T   T   T
  0.3011899950000014  0.1164099980000017  0.0016000000000034   F   F   F
  0.3095700240000028  0.7886100039999988  0.0893600010000029   F   F   F
  0.3336670851484561  0.5075405058933770  0.1750322928036802   T   T   T
  0.3519983386130924  0.1834896275432434  0.2637036771751098   T   T   T
  0.8057299720000017  0.0390500029999998  0.0533899989999966   F   F   F
  0.8111099859999982  0.7014499889999968  0.1424400100000014   F   F   F
  0.8355983251432101  0.3407100890766023  0.2260236946808704   T   T   T
  0.8520453088136665 -0.0035761351938422  0.3093469218394792   T   T   T
  0.6198724810817277  0.0082574065251129  0.3334359117765278   T   T   T
  0.5931568084899029  0.3301450654560142  0.3264860404818882   T   T   T
```

※ (101) -H⁺-H⁺

```
1.00000000000000
      5.5226998328999999    0.0000000000000000    0.0000000000000000
      0.0000000000000000    4.6612000465000003    0.0000000000000000
      0.0000000000000000    0.0000000000000000   29.3530006408999995
   Ti   O   H
     8   16    2
Selective dynamics
Direct
  0.0231299989999982  0.3380999910000000  0.0276799999999966   F   F   F
  0.9956499520000008  0.9953600139999992  0.1147900030000031   F   F   F
  0.9893154934300281  0.6874982160464927  0.2020638435250713   T   T   T
```

0.0103273647415964	0.2813095176669463	0.2833660452326817	T	T	T
0.4768699819999966	0.8381000170000021	0.0276799999999966	F	F	F
0.5043500049999992	0.4953599629999985	0.1147900030000031	F	F	F
0.5113245372966442	0.1876271715267699	0.2021055790048440	T	T	T
0.4911781791341317	0.7796265093388678	0.2833120185673110	T	T	T
0.6942700279999983	0.5390499680000005	0.0533899989999966	F	F	F
0.6888899710000018	0.2014500020000014	0.1424400100000014	F	F	F
0.6614141607878694	0.8356734547282445	0.2241130222598008	T	T	T
0.7212643820025448	0.5126665882576854	0.3211052926802438	T	T	T
0.1988100049999986	0.6164100230000003	0.0016000000000034	F	F	F
0.1904299979999990	0.2886100039999988	0.0893600010000029	F	F	F
0.1677672385154986	0.0024381722615969	0.1754921489313303	T	T	T
0.1354760306296870	0.7050570303840259	0.2660342467240259	T	T	T
0.3011899950000014	0.1164099980000017	0.0016000000000034	F	F	F
0.3095700240000028	0.7886100039999988	0.0893600010000029	F	F	F
0.3320573023291198	0.5022152312528652	0.1755588269366501	T	T	T
0.3658330278857834	0.2051166348790354	0.2661467692898015	T	T	T
0.8057299720000017	0.0390500029999998	0.0533899989999966	F	F	F
0.8111099859999982	0.7014499889999968	0.1424400100000014	F	F	F
0.8390533392369210	0.3358237442262950	0.2242147509364948	T	T	T
0.7793375628906906	0.0122859695963971	0.3205282630097958	T	T	T
0.8346579793999219	0.8296689283694365	0.3338805243138078	T	T	T
0.6655421903756396	0.3277749219851417	0.3336690210056010	T	T	T

© 2019 by the authors. Submitted for possible open access publication under the terms and conditions of the Creative Commons Attribution (CC BY) license (http://creativecommons.org/licenses/by/4.0/).
